# Optimizing Nickel(II) Complex Catalysts for High-Yield Oligomerization of Cyclohexyl Isocyanide

**DOI:** 10.1021/acs.inorgchem.4c04516

**Published:** 2025-03-03

**Authors:** Marta Pawlak, Joanna Drzeżdżon, Katarzyna N. Jarzembska, Dagmara Jacewicz

**Affiliations:** † Department of Environmental Technology, Faculty of Chemistry, University of Gdansk, Wita Stwosza 63, Gdansk 80-308, Poland; ‡ Department of Chemistry, University of Warsaw, Żwirki i Wigury 101, Warsaw 02-089, Poland

## Abstract

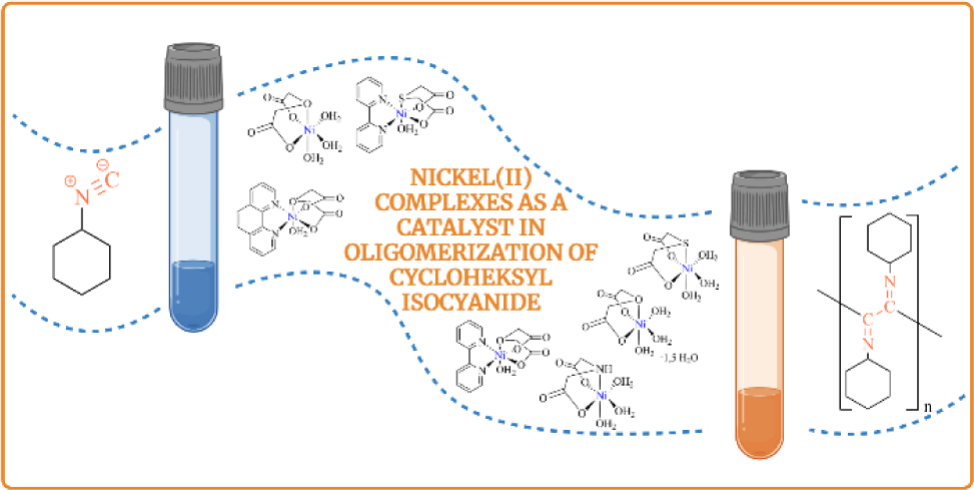

Isocyanides, due to the divalent carbon atom present in their structure, are among the most reactive groups of compounds in organic chemistry. Unfortunately, although according to the literature they do not have acute toxicity, they have a very unpleasant odor that makes it difficult to collaborate with them. However, despite the properties mentioned, reactions of isocyanides often lead to a variety of functional materials. In this article, we present a modified method for obtaining isocyanide-based polymers that significantly reduce the release of their hazardous vapors into the environment. For the study, a series of nickel(II) metal ion complex compounds containing organic ligands (e.g., 2,2′-bipyridyl, 1,10-phenanthroline, and diglycolate anion) were synthesized and used as catalysts in the oligomerization reaction of cyclohexyl isocyanide. The obtained oligomers were subjected to quantitative and qualitative physicochemical analyses (FT-IR, MALDI-TOF-MS, TGA/DSC, and DSC), which confirmed their structure and thermal properties. Reaction yields ranged from moderate (8–52%) to extremely high (94%) for a single catalyst. The synthesized catalytic systems are new, previously undescribed isocyanide oligomerization catalysts, which successfully led to the synthesis of poly(cyclohexyl isocyanide) and allowed us to obtain materials that can be used to produce many useful polymeric materials.

## Abstract 2

Our article presents a modified method for obtaining isocyanide-based polymers that results in a reduction in the release of hazardous isocyanide vapors into the environment. During the study, nickel(II) complex compounds were used as catalysts in the oligomerization reaction of cyclohexyl isocyanide. The catalytic systems presented are new, previously undescribed isocyanide oligomerization catalysts that have successfully synthesized poly(cyclohexyl isocyanide) and have yielded materials that can be used to produce many useful polymeric materials.

## Introduction

Isocyanides are one of the most interesting groups of compounds found in organic chemistry, and they are attracting the attention of chemists because of their original structure, more specifically, the divalent carbon atom present in their structure.^1−3^ Despite this advantage, isocyanides are unfortunately perceived as toxic compounds due to their unpleasant odor. Although according to the literature, most isocyanides do not exhibit acute toxicity, the unpleasant odor makes working with this group of compounds much more difficult and dangerous.

**Figure 1:**
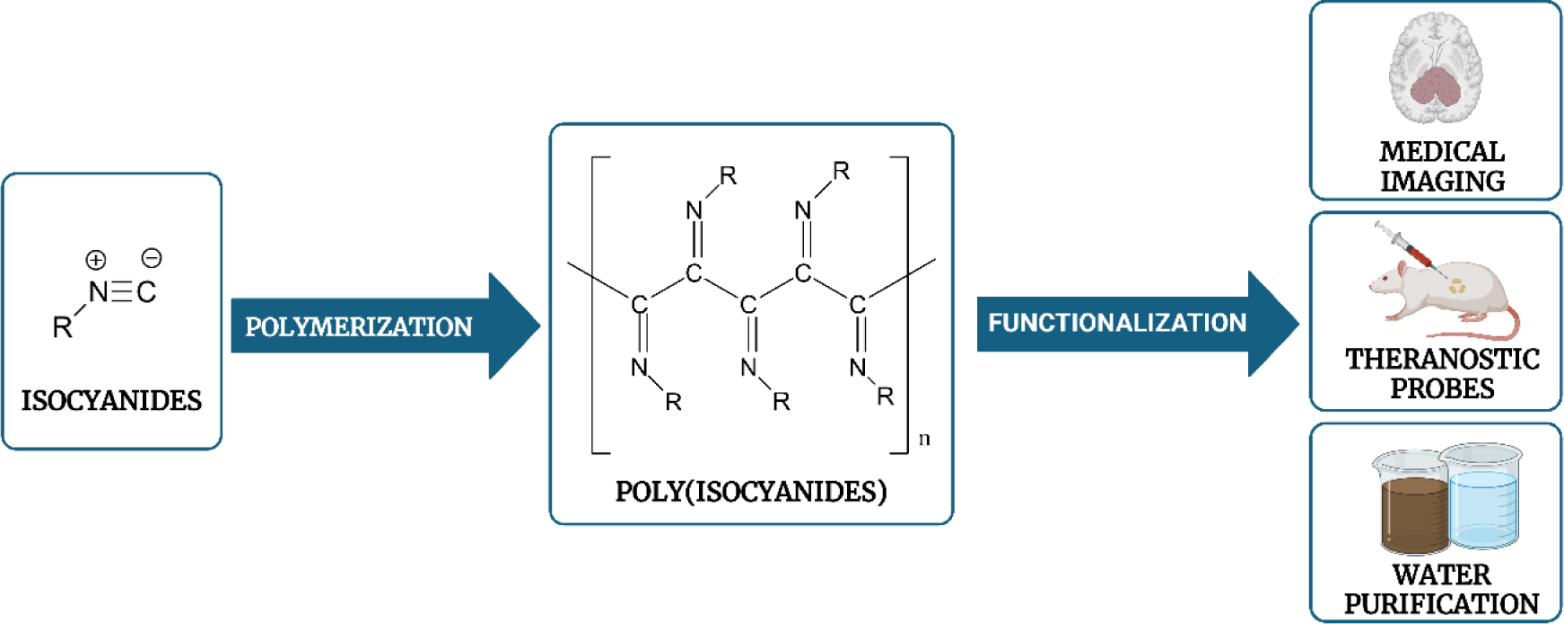
Schematic of the use of isocyanides in the polymerization reaction.

One of the reactions in which isocyanides can participate is their polymerization, which can lead to many functional materials.^4−6^ In addition, isocyanide-based polymers (IBPs) exhibit unique chemical, biological, and optical properties, which make them applicable to both science and industry and to the development of some functional materials,^4−6^ e.g., a series of isocyanide-based polymers exhibiting luminescent properties that can be used in medical imaging^5^ or as theranostic probes^7^ (Figure 1).

The research difficulty in isocyanide polymerization was initiated by Millich in 1975.^8^ Subsequent research on the polymerization of isocyanides focused on the introduction of catalysts and initiators into the reaction.^9^ Nolte^10^ proved to be a pioneer in further research on isocyanide polymerization and the use of metal ions in this reaction.

The catalysts for isocyanide oligomerization presented until now in the literature are mainly based on simple nickel(II) salts, e.g., NiCl_2_, Ni(acac)_2_.^11^ Currently, there is a white spot in the literature on the topic of using other nickel complex compounds in isocyanide oligomerization processes. However, complex compounds based on nickel(II) ions are often used in oligomerization processes of other monomers (e.g., olefins)^12^ and in these processes; they show high values of catalytic activity. In addition, the wide variety of compounds that can be used as ligands in the synthesis of catalysts can allow for the structure of compounds acting as catalysts to be precisely tailored to the needs of a particular process.

There are also several disadvantages to using coordination compounds in this process. First, already at the stage of designing the synthesis of these compounds, it is necessary to consider the appropriate choice of ligands. The ligands within the complexes’ structure must not block or obstruct the active sites on the nickel(II) ions and should not introduce significant steric hindrance that could impede the monomer’s access to the active site.^13,14^

The selection of a suitable compound acting as a ligand is extremely difficult; in the presented study, it was checked whether it is possible to extend the remarkable catalytic properties of nickel(II) complex compounds by another oligomerization process. A study was conducted during which the classical isocyanide oligomerization process was modified by using catalysts of a different type. The specially selected structure of the compounds used in the study can result in more efficient catalysis of the isocyanide oligomerization process, which translates into a shorter process running time and higher yields of the obtained products. In addition, the process was transferred to a sealed reactor using the minimum required quantities of hazardous substances, resulting in a significant reduction in the exposure of the synthetic chemist to their odor, limiting the release of hazardous vapors into the environment.

Polycarboxylate nickel(II) compounds were chosen as catalysts because the nickel systems used in isocyanide oligomerization processes are easy to configure and efficient.^15^ In addition, the use of catalytic systems based on nickel(II) ions provides mild reaction conditions (polar solvents, *T* = 25 °C), and the salts used in the synthesis of catalysts are cheap and readily available on the market.^15,16^ Polycarboxylate nickel(II) compounds are readily soluble in water, odorless, and do not cause irritation upon human contact, and these compounds can also exhibit antioxidant, antimicrobial, and antifungal properties, which is a significant contrast to the slightly toxic isocyanides.

In this study, we have synthesized seven nickel(II) metal ion complex compounds and used them as catalysts in the oligomerization of cyclohexyl isocyanide to evaluate their ability to form poly(isocyanide) oligomers and to see with what efficiency they would lead to the desired oligomers. When conducting the oligomerization reaction of cyclohexyl isocyanide using nickel(II) complex compounds, it is crucial to maintain appropriate conditions such as high purity of the complexes used and all reagents used, conduct the reaction under a nitrogen atmosphere, and control the reaction temperature, which should not exceed 28 °C. Maintaining the right conditions throughout the process will ensure that oligomers are obtained with high yields and selectivity.

To date, only the use of simple nickel(II) compounds (e.g., Ni(acac)_2_) as transition metal-based catalysts in the oligomerization and polymerization of isocyanides has been studied in the literature. Although the presented compounds allow for achieving the desired polymers with yields close to 100% they do not allow the control of the structure and properties of the obtained materials, nor for studying the reaction mechanism in detail. The study to date also does not provide information on the effect of catalyst structure on polymerization yields. Our research will enrich the literature by describing the use of more structurally complex nickel(II) coordination compounds and describing the effect of their structure on the catalytic activity and performance of the polymerization and oligomerization process of cyclohexyl isocyanide. The reports are also the basis for further analysis of the effect of catalyst structure on the properties of the materials obtained, and in the future, the possibility of creating materials with tailored structures. They also serve as a good introduction to begin more in-depth studies on the suggested mechanism of catalysis. Moreover, with the knowledge gained during the study, it is possible to further develop an understanding of the isocyanide oligomerization process using *N*,*N*-donor nickel(II) complex compounds. In the literature,^17,18^ many examples of complexes showing high catalytic activities in the oligomerization processes of other monomers are described. Future research directions should also include testing the activity of other coordination compounds described in the literature and searching among them for a highly active catalyst, which will lead to high yields under safe conditions and allow for the control of the properties and structure of the obtained oligomers.

## Experimental Section

### Chemicals

All chemical reagents were purchased from Merck (Darmstadt, Germany), and according to the data from the company, their purity was: diglycolic acid (98%); sodium carbonate (99.9%); nickel(II) chloride hexahydrate (99.9%); acetone (99.5%); diethyl ether (99.0%); 2,2′-bipyridine (99%); 1,10-phenanthroline monohydrate (99.5%); thiodiacetic acid (98%); nickel(II) acetate tetrahydrate (98%); iminodiacetic acid (98%); cyclohexyl isocyanide (98%); ethanol (96%); methanol (99%).

### Synthesis of Complex Compounds

The synthesis of the complex compounds [Ni(TDA)(H_2_O)_3_], [Ni(ODA)(H_2_O)_3_]·1.5 H_2_O, [Ni(IDA)(H_2_O)_3_], [Ni(TDA)(phen)(H_2_O)], [Ni(TDA)(bipy)(H_2_O)]·4 H_2_O, [Ni(ODA)(phen)(H_2_O)]·1.5 H_2_O, and [Ni(ODA)(bipy)(H_2_O)]·2.5 H_2_O was performed following mildly modified procedures reported in the literature.^19−23^

Prior to oligomerization, the resulting coordination compounds were thoroughly dried to remove excess water molecules present outside the coordination sphere.

### The Oligomerization Processes

The isocyanide oligomerization process was conducted based on the isocyanide polymerization procedure available in the literature.^11^

The oligomerization process was conducted under inert gas conditions (nitrogen) in a glass cell in a water bath at room temperature. Initially, 0.07 mmol of the nickel(II) metal ion complex compound was dissolved in ethanol (4 mL). Then, 0.512 mL of cyclohexyl isocyanide was added, and the process of isocyanide oligomerization began; the reaction was conducted for 2 h, during which time the formation of a creamy-white powder could be observed. In the final step, the nitrogen supply was cut off, and the resulting products were washed with methanol (2 mL) to remove residual of catalysts. Subsequently, the resulting oligomers were separated from the reaction mixture by gravity drainage under a switched extractor with exceptional care. The resulting products were dried to a solid mass.

### Infrared (FTIR) Spectra

The IR spectra were recorded in the 4500–500 cm^–1^ range on a KBr pastille. DLATGS (Branch Ü Bellingen, Germany) was used as a detector. The IFS66 apparatus by BRUKER (Branch Ü Bellingen, Germany) had a resolution of 0.12 cm^–1^.

### Flight Mass Spectrometry (MALDI-TOF-MS) Spectra

MALDI-TOF-MS spectra were rerecorded on a BRUKER autoflex maX (Branch Ü Bellingen, Germany) using 2,5-dihydroxybenzoic acid (DHB) as a matrix during the experiments. The measurement device is equipped with a smartbeam-II solid-state laser.

### Simultaneous TGA/DSC Thermal Analysis

The TGA/DSC measurements were performed using a Mettler-Toledo TGA/DSC STAR^e^ system at a heating rate of 10 °C/min under a dry N_2_ atmosphere and at a constant flow (60 mL/min) over the 25–1000 °C temperature range. The obtained data were analyzed by using the STARe software provided by Mettler-Toledo. Prior to the TGA/DSC measurements, each sample was accurately weighed in a standard 40 μL alumina crucible using a Mettler-Toledo XS105 DualRange balance.

### Differential Scanning Calorimetry (DSC)

The DSC measurements were performed using a Mettler-Toledo DSC1 STAR^e^ system at a heating rate of 10 °C/min under a dry N_2_ atmosphere and at a constant flow (50 mL/min) over the 25–600 °C temperature range. The obtained data were analyzed using the STARe software provided by Mettler-Toledo. Prior to the DSC measurements, each sample was accurately weighed in a standard 40 μL aluminum crucible using a Mettler-Toledo XS105 DualRange balance.

## Results and Discussion

An especially important initial part of the study was the selection of suitable compounds to function as catalysts. It was necessary to match complexes with the appropriate ligand and metal ion, which, through adaptability, affects the structure and properties of the final product. Even a minor change in the structure of the complex compound used as a catalyst can lead to significant changes in its catalytic activity and the efficiency of the oligomerization process.^13^ Improperly selected ligands can affect a significant reduction in the catalytic activity of the compounds, cause an increase in reaction running time, and reduce the efficiency with which oligomers are obtained.^13,14^ In addition, isocyanides, due to their structure, are extremely reactive compounds, so during the process of synthesizing a complex, it should also be taken into account that the ligands in the catalyst structure do not react with the added monomer, leading to the formation of a different end product or the production of byproducts.^24^

The nickel(II) ions were chosen as the metal center because it had already been proven that almost any isocyanide can be polymerized using nickel(II) compounds.^16^ A study was also carried out in which complexes of other metals (cobalt(II), palladium(II), copper(II), platinum(II), manganese(II), cadmium(II)) were tested in the oligomerization of ethyl isocyanide (Table 1), and it was proven that a compound containing nickel(II) in the metal center leads to the highest yield and additionally provides milder conditions (lower process temperature and milder solvents).^11^ Moreover, some of them are very toxic and difficult to access, and they require more difficult reaction conditions.^16^ In addition, catalytic systems based on palladium(II) ions are more difficult to reduce when conducting the reaction, and catalysis using them is much slower than when using nickel(II) compounds.^25^ Moreover, the use of Ni(II) compounds provides stronger bonds and will reduce liability, providing control throughout the reaction.^25^

**Table 1:**
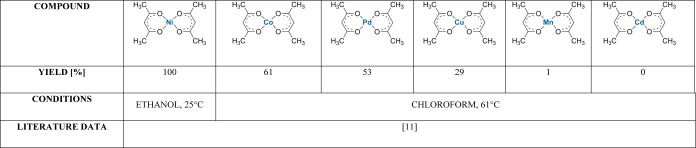
Results of Ethyl Isocyanide Polymerization by Different Transition Metal Catalysts

**Figure 2:**
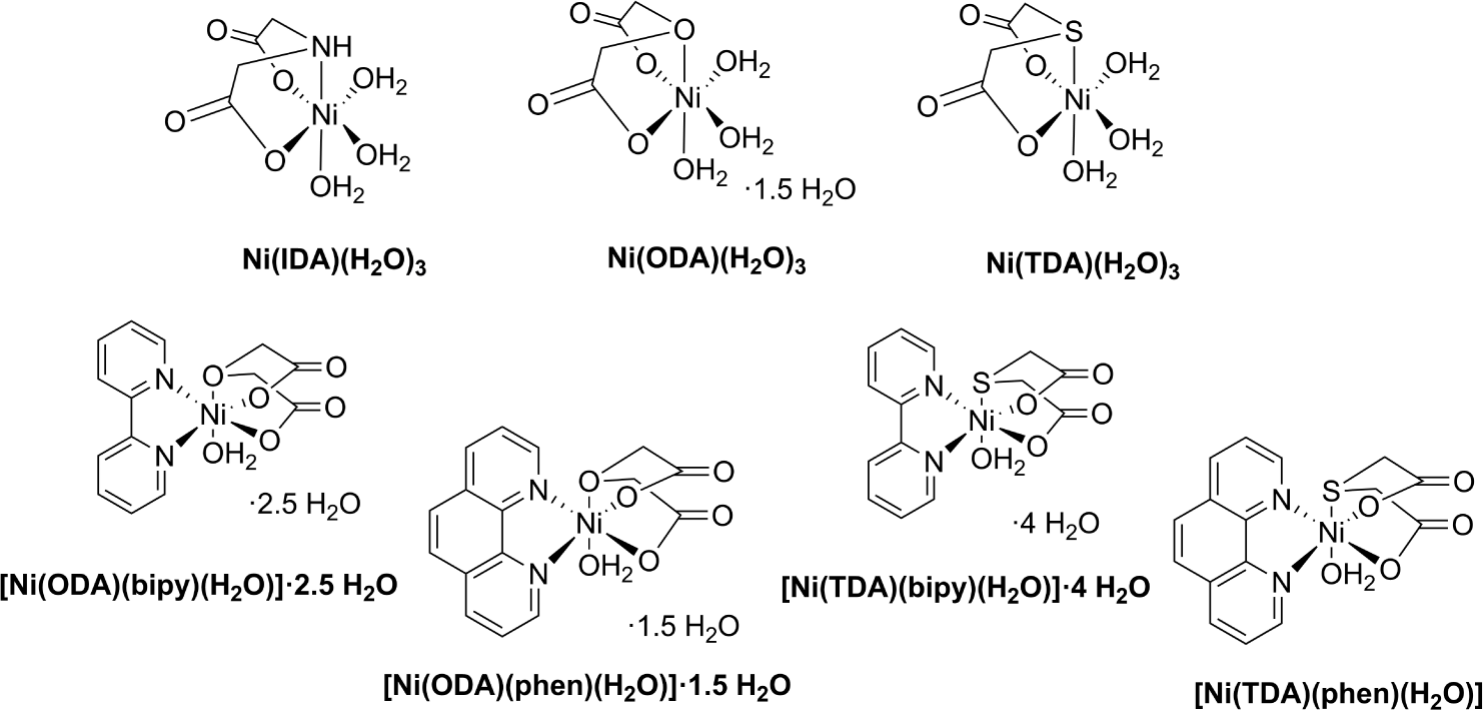
Synthesized nickel(II) metal ion complex compounds, where 2,2′-bipyridine (bipy), 1,10-phenanthroline (phen), diglycolic acid (ODA), thiodiacetic acid (TDA), and iminodiacetic acid (IDA).

After careful analysis and selection, seven nickel (II) complex compounds containing organic ligands, such as 2,2′-bipyridine (bipy), 1,10-phenanthroline (phen), diglycolic acid (ODA), thiodiacetic acid (TDA), and iminodiacetic acid (IDA) were synthesized (Figure 2).

Initially, the chemical composition of the obtained complexes [Ni(TDA)(H_2_O)_3_], [Ni(ODA)(H_2_O)_3_]·1.5 H_2_O, [Ni(IDA)(H_2_O)_3_], [Ni(TDA)(phen)(H_2_O)], [Ni(TDA)(bipy)(H_2_O)]·4 H_2_O, [Ni(ODA)(phen)(H_2_O)]·1.5 H_2_O, and [Ni(ODA)(bipy)(H_2_O)]·2.5 H_2_O was determined by elemental analysis studies, which made it possible to determine the percentage of carbon, hydrogen, sulfur, and nitrogen in the obtained complexes and to compare the obtained values with those calculated based on theoretical calculations. The results of the elemental analysis are presented in Table S1,In addition the compounds were also examined by spectroscopic methods, using UV–vis analysis (aqueous solutions and solid-state) and FT-IR technique, to confirm their structure. The analyses yielded spectra that confirmed the presence of bands characteristic of the structure of the synthesized coordination compounds and further confirmed the purity of the obtained compounds. UV–VIS analysis was also conducted for the substrates used, and the UV–VIS spectra presented for these compounds coincide with those reported in the literature.^26−31^

**Figure 3:**
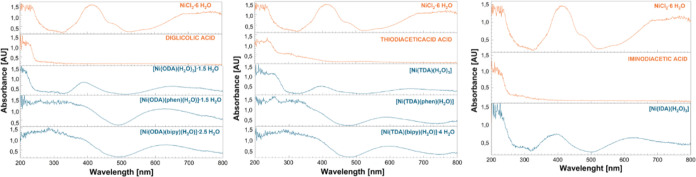
Comparison of DRS/UV–spVIS spectra of complex compounds and diglycolate, iminodiacetate, and thiodiacetate.

**Figure 4:**
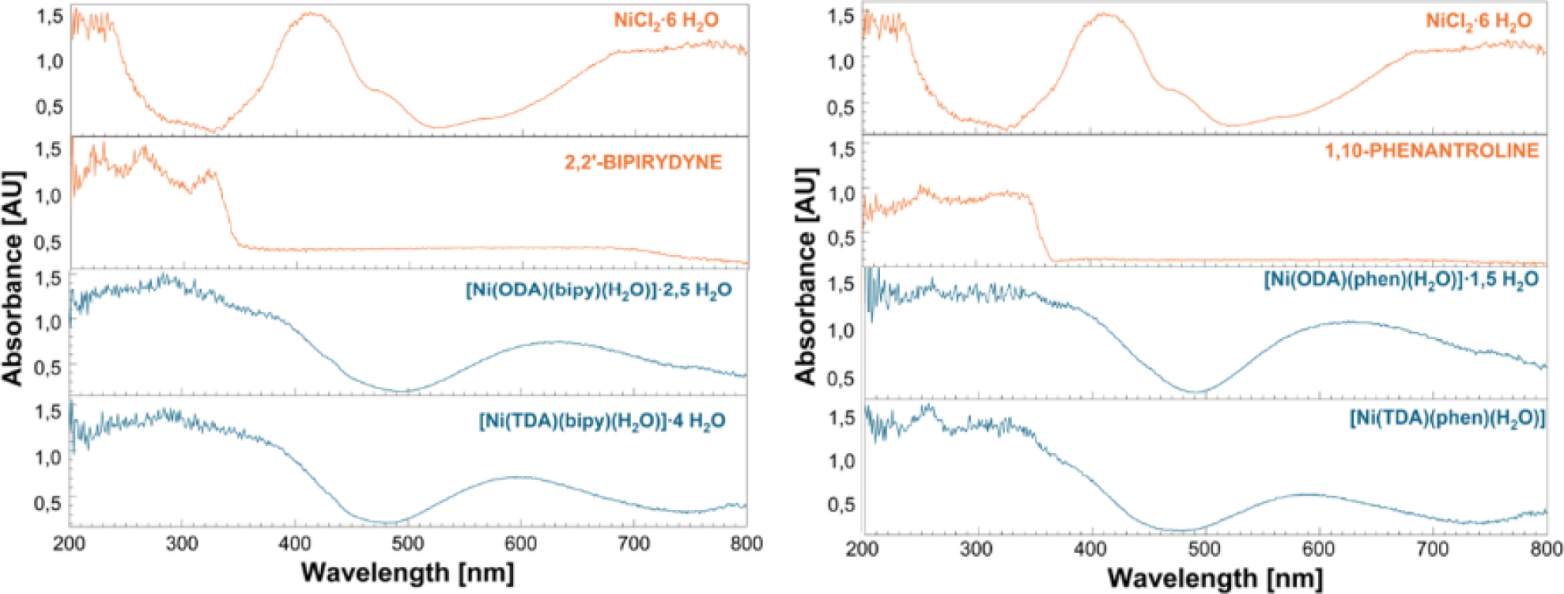
Comparison of DRS/UV– VIS spectra of complex compounds and 1,10-phenanthroline and 2,2′-bipyridyl ligands.

DRS/UV-VIS analysis of the f resulting complexes and the substrates used for their synthesis (Figures 3 and 4), followed by their comparison, made it possible to assign the origin of the bands obtained from the spectra. A detailed description of the DRS/UV–cVIS spectra is available in the Supporting Information.

Thanks to the obtained UV–VIS spectra, it is also possible to determine the electron transitions between the metallic center and the ligands in the analyzed compounds. When analyzing the spectra of NiCl_2_·6H_2_O and all complex compounds, hypsochromic shifts are observed in the spectra of the complexes relative to the bands present in the spectrum of nickel(II) chloride. Such shifts and the reduced intensity of bands originating from nickel(II) ions indicate the occurrence of electron transitions. In the spectra of the compounds [Ni(TDA)(H_2_O)_3_], [Ni(ODA)(H_2_O)_3_]·1.5H_2_O, and [Ni(IDA)(H_2_O)_3_], a high intensity of ligand-derived bands can be observed. This, combined with the fact that the compounds contain acetic acid-based ligands, which due to the presence of a free electron pair in their structure are included in the group of donor ligands, indicates the presence of LMCT (ligand–metal charge transfer) transitions and electron transfer from the ligand structure to the metal. While for compounds [Ni(ODA)(bipy)(H_2_O)]·2.5 H_2_O, [Ni(ODA)(phen)(H_2_O)]·1.5 H_2_O, [Ni(TDA)(bipy)(H_2_O)]·4 H_2_O, and [Ni(TDA)(phen)H_2_O], in which highly acceptor polypyridine ligands are attached, we observe slightly higher intensity and jagged bands, which may indicate the presence of MLCT (*metal–ligand charge transfer)* transitions. In addition, the combination of highly acceptor polypyridine ligands and donor ligands results in the occurrence of LLCT (*ligand-to-ligand charge transfer*) transitions, which we also observe in the spectra in Figure 4.

The structure of the synthesized complex compounds was also confirmed by performing FT-IR spectra. The FT-IR spectra with descriptions are available in the Supporting Information. The content and spectroscopic data for the synthesized complex compounds are in agreement with previously reported values (Table S3).

The synthesized nickel(II) complex compounds were used as catalysts in a slightly modified process of oligomerization of cyclohexyl isocyanide. The most notable modification, compared to the existing isocyanide polymerization process, is the use of a different type of catalyst. The catalytic systems used in the study are coordination compounds whose structure consists of nickel(II) ions in the coordination center and −N, −O, and −S donor ligands in the coordination sphere. Additionally, the entire process was conducted in a manner that reduced exposure to hazardous isocyanide vapors and limited their release into the environment. The process was conducted in a vial, tightly sealed with a cork, and additionally protected with a parafilm, which significantly reduced the release of dangerous vapors outside the reactor, whereas the necessary quantities of isocyanides needed for the reaction were introduced directly into the reactor using a syringe. The oligomerization products were obtained in the form of yellow-cream powders (Figure S7), which, according to the available literature,^11^ are characteristic of poly(cyclohexyl isocyanide).

**Figure 5:**
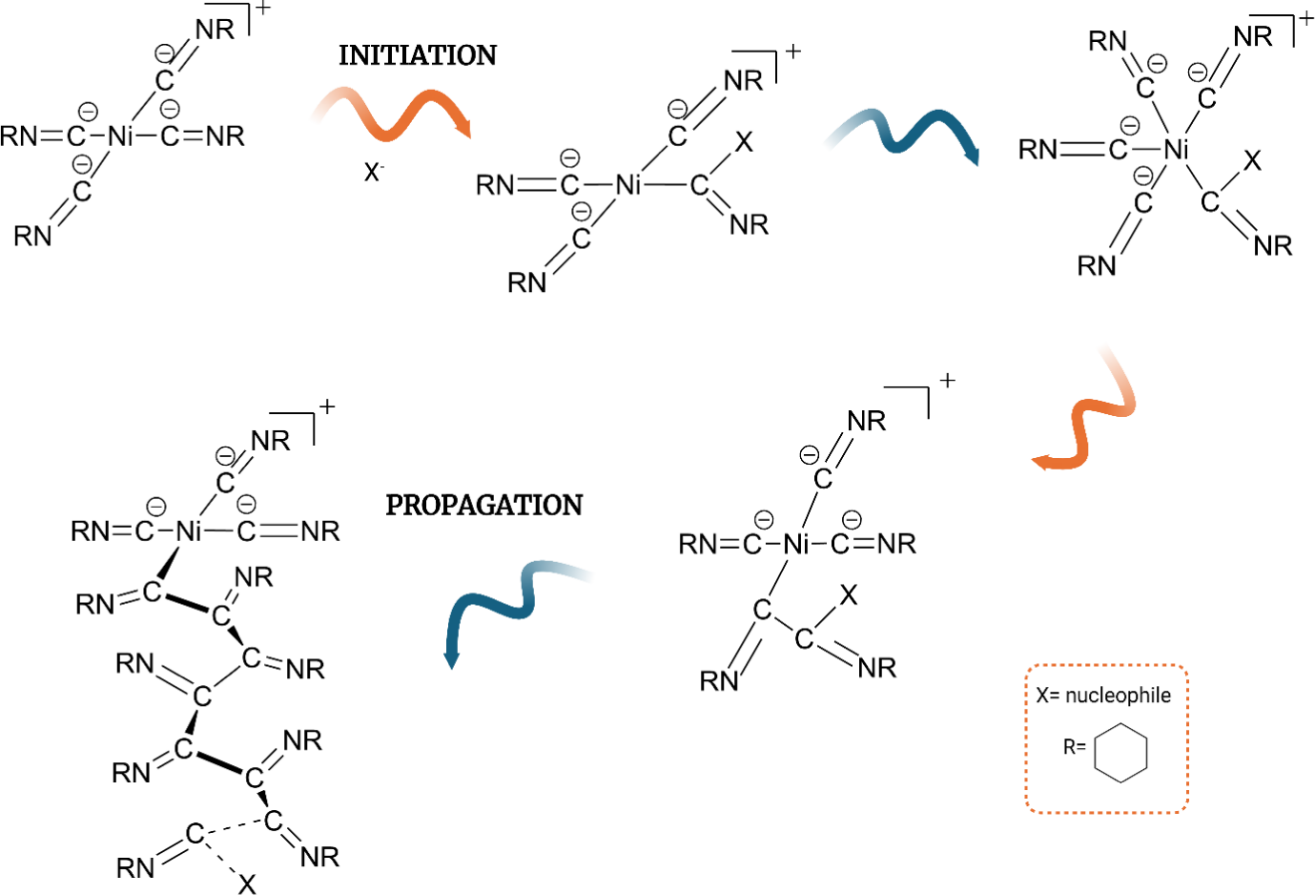
Suggested mechanism for cyclohexyl isocyanide oligomerization catalyzed by nickel(II) ions.

The mechanism of nickel-catalyzed isocyanide polymerization has been studied and presented in the literature by Drenth and Nolte.^32^ Based on the available literature, it can be suggested that the nickel-catalyzed oligomerization of cyclohexyl isocyanide, presented in this study, also follows this mechanism (Figure 5).

This mechanism has been referred to as the “merry-go-round” mechanism. In the first step, a cationic nickel(II) tetrakis(isocyanide)complex is formed before initiation. The nucleophile then attacks one of the carbon atoms of the isocyanide, generating α-iminiomethyl-nickel(II) complexes. In the next step, the fifth isocyanide is coordinated to the nickel center, and the iminomethyl group migrates to the adjacent isocyanate carbon atom, forming a dimeric intermediate. In the final phase, this step is repeated to form a poly(cyclohexyl isocyanide).

The best oligomerization yield (94%) was achieved using [Ni(ODA)(bipy)(H_2_O)]·2.5 H_2_O as a catalyst. A high efficiency (52%) was also achieved by shuttling [Ni(ODA)(H_2_O)_3_]·1.5 H_2_O. The efficiency of the process using the other catalysts [Ni(ODA)(phen)(H_2_O)]·1.5 H_2_O (8%), [Ni(TDA)(H_2_O)_3_] (10%), [Ni(TDA)(phen)H_2_O] (22%), [Ni(IDA)(H_2_O)_3_] (23%), and [Ni(TDA)(bipy)(H_2_O)]·4 H_2_O (27%) is rather low and can be classified as unsatisfactory. Large differences in process efficiency also coincide with differences in catalytic activity due to changes in the structures of the catalysts used. The exact effect of structure on catalytic activity and efficiency will be described later in the manuscript.

**Figure 6:**
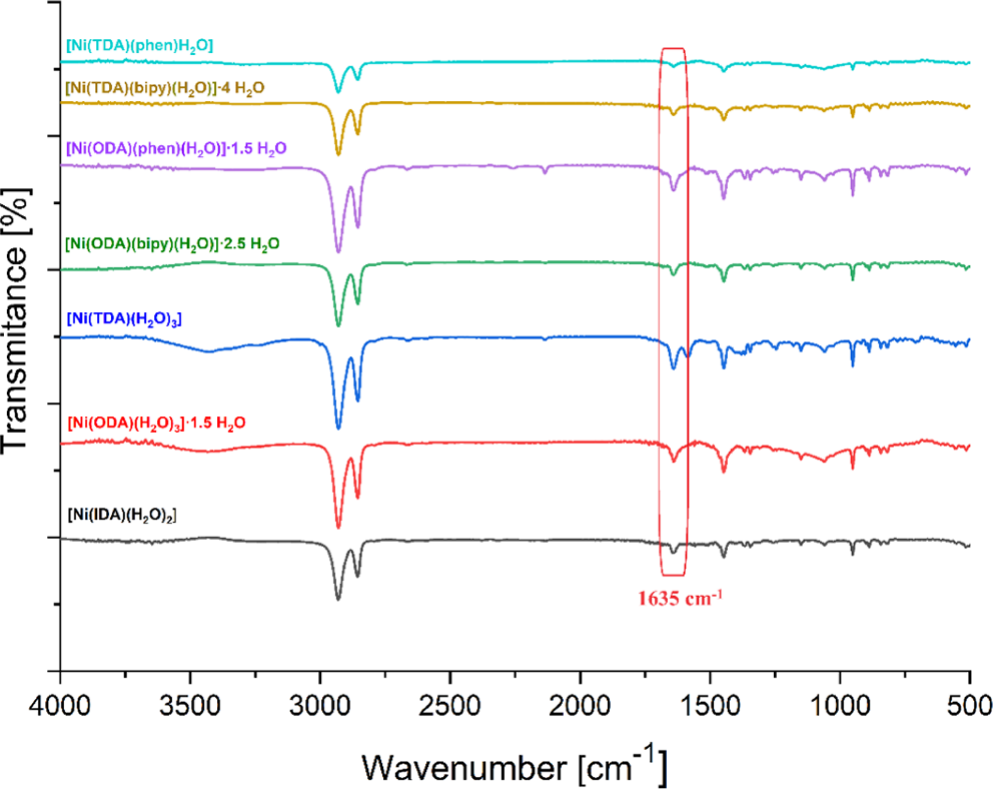
Comparison of FT-IR spectra for all oligomerization products.

The structure of the resulting oligomerization products was confirmed by FT-IR. Comparing the FT-IR spectra for each product (Figure 6), we can observe the appearance of a band at 1635 cm^–1^, which is characteristic of the N=C bond in the formation of poly(cyclohexyl isocyanide). In addition, the spectra also show the disappearance of a band at 2180–2120 cm^–1^, which is a band characteristic of the N≡C bond found in isocyanides. The disappearance of the band indicates the breaking of the bond between nitrogen and carbon while also indicating the formation of the oligomer. Bands at wavelengths in the 2900–2800 cm^–1^ range correspond to the stretching vibrations of the CH_2_ group in the alkene cyclic chain, while bands at 1475–1450 cm^–1^ are characteristic of the deformation vibrations of the CH_2_ group also present in the cyclic ring of cyclohexyl isocyanide. Bands in the 1000–800 cm^–1^ range, on the other hand, are characteristic of the C–N bond.

MALDI-TOF-MS analysis showed that the resulting oligomerization products are macromolecular compounds formed as a mixture of oligomers consisting of fragments with different chain lengths. The results show that the resulting oligomers consist of short chains with lengths ranging from 1 to a maximum of 7 mer. MALDI-TOF-MS spectra for the individual oligomerization products are available in (Figures S8–S14).

The glass transition temperature, crystallization temperature, and melting point of the analyzed oligomers were determined by DSC analysis, and the obtained data are shown in Table 2. DSC curves for all analyzed samples are available in Figures S15–S21. DSC analysis allowed for the study of thermal effects occurring during heating. In the resulting DSC curves, the occurrence of both exothermic processes (crystallization) and endothermic processes (melting, change of state) can be observed. According to the literature, poly(cyclohexyl isocyanide) melts above 329 °C.^8^ Melting point values for all samples are higher, close to each other, and oscillate in the 420–441 °C range, which is very high compared to the well-known polymers [Tm_PE_ = 126–135 °C, Tm_PP_ = 160–170 °C, Tm_PU_ = 120–250 °C (PE-polyethylene, PP-polypropylene, PU- polyurethanes)]. Equally close to each other are the crystallization temperatures located in the range of 313–322 °C. By analyzing the DSC curves, it is also possible to determine the glass transition temperature, which oscillates in the 157–193 °C range for all samples. The occurrence of the glass transition temperature indicates the occurrence of the transition from the glassy state to the elastic state and is characteristic of oligomers of semicrystalline or amorphous structure.

**Table 2:** Temperatures of Oligomerization Products Determined by DSC Analysis^a^

Catalysts	Tg [°C]	Tc [°C]	Tm [°C]
[Ni(IDA)(H_2_O)_3_]	140	326	419
[Ni(ODA)(H_2_O)_3_]·1.5 H_2_O	182	327	435
[Ni(TDA)(H_2_O)_3_]	157	313	430
[Ni(ODA)(bipy)(H_2_O)]·2.5 H_2_O	182	323	429
[Ni(ODA)(phen)(H_2_O)]·1.5 H_2_O	184	328	439
[Ni(TDA)(bipy)(H_2_O)]·4 H_2_O	193	322	441
[Ni(TDA)(phen)H_2_O]	169	327	443

a Tg- glass transition; Tc-crystallization temperature; Tm- melting temperature

TGA combined with DSC analysis was performed to determine the thermal stability of the samples and the decay rates of the products. TGA curves for each product can be found in Figures S22–S28. A characteristic feature of polyisocyanide homopolymers is a large initial weight loss reaching about 50%.^8^ In the analyzed curves, the largest weight loss occurs in the 230–370 °C temperature range and is about 40% for all analyzed samples. The second large mass loss of about 30% can be observed in the temperature range of 370–520 °C. The recorded theremogravimetric curves and the data presented therein, regarding the temperature range and mass loss, coincide with the curved^33^ The rate of degradation for the analyzed products ranged from 0.09 to 0.13 mg/min. Thermal degradation of most well-known polymers starts at 150–200 °C and ends below 400 °C;^34^ for the analyzed samples, this range is higher (230–530 °C). The literature reports that poly(isocyanides) does not undergo thermal decomposition up to 300 °C and that the weight losses stretch over a large temperature range.^8,11,33^ Thermogravimetric analysis for all analyzed samples presented a wide range of decomposition temperatures and a pronounced mass loss only above 300 °C. Thermal analysis of the obtained oligomers and comparison of the data with literature data allow us to conclude that the obtained oligomers have high thermal stability, which is also confirmed by their high melting point.

To date, the literature has not reported a method for calculating catalytic activity in the oligomerization of isocyanides, while the activity of catalytic systems is compared with the yield of the obtained product. However, standard calculations of catalytic activity, in oligomerization processes of other monomers, are based on the mass of oligomer obtained, the duration and conditions of the process, and the amount of catalyst used.

Using the formula presented (eq 1) and the necessary data, the catalytic activity of the systems used in the study was calculated. The calculated values of catalytic activity in the oligomerization of isocyanides are presented in Table 3.
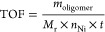
1where *m*_oligomer_ is the weight of oligomer [g], *M*_r_ is the molar weight of cyclohexyl isocyanide [109 g/mol], *n*_Ni_ is the amount of catalyst [mol], and *t* is the reaction time [h]

**Table 3:** Results of the Calculated Catalytic Activity

Entry	Complex compound	Weight of the resulting oligomer [g]	Number of moles of catalyst [mol]	Duration of the process [h]	TOF [g mol^–1^ h^–1^]
1	[Ni(IDA)(H_2_O)_3_]	0.1060	7 × 10^–5^	2	757.1
2	[Ni(ODA)(H_2_O)_3_]·1.5 H_2_O	0.2350			1678.6
3	[Ni(TDA)(H_2_O)_3_]	0.0457			326.4
4	[Ni(ODA)(bipy)(H_2_O)]·2.5 H_2_O	0.4047			2890.7
5	[Ni(ODA)(phen)(H_2_O)]·1.5 H_2_O	0.0370			264.3
6	[Ni(TDA)(bipy)(H_2_O)]·4 H_2_O	0.1221			872.2
7	[Ni(TDA)(phen)H_2_O]	0.0999			713.6

Analyzing the values obtained, it is possible to determine the relationship between the structure of the main ligands and accessory ligands and the values of catalytic activity. Comparing the effect of the main ligand on the values of catalytic activity in the compounds [Ni(IDA)(H_2_O)_3_], [Ni(ODA)(H_2_O)_3_]·1.5 H_2_O, and [Ni(TDA)(H_2_O)_3_], it can be seen that the highest value was achieved with the use of complexes containing diglycolic acid as the main ligand (Table 3, entry 2), while the lowest value was achieved by the use of thiodiacetic acid as the main ligand (Table 3, entry 3). Such a discovery is due to a change in the stability of the complex caused by a change in the donor atom in the ligand. Oxygen has the lowest electronegativity among all of the donor atoms. Ligands in which the donor atom is this element are the most stable and provide the highest values of catalytic activity, and as the electronegativity of the donor atom increases, the stability of the compounds decreases, and consequently, their ability to catalyze the oligomerization reaction of cyclohexyl isocyanide also decreases. Comparing the activity of compounds [Ni(ODA)(H_2_O)_3_]·1.5 H_2_O, [Ni(ODA)(bipy)(H_2_O)]·2.5 H_2_O, and [Ni(ODA)(phen)(H_2_O)]·1.5 H_2_O], it can be concluded that the introduction of an accessory ligand in the structure of the compound in the form of 2,2′-bipyridyl and 1,10-phenanthroline also causes a rapid change in catalytic activity. In addition, analyzing the activity of compounds [Ni(ODA)(bipy)(H_2_O)]·2.5 H_2_O, [Ni(ODA)(phen)(H_2_O)]·1.5 H_2_O, [Ni(TDA)(bipy)(H_2_O)]·4 H_2_O, and [Ni(TDA)(phen)H_2_O] it can be seen that compounds containing 2,2′-bipyridyl in their structure showed higher values of catalytic activity than compounds containing the ligand in the form of 1,10-phenanthroline (Table 3, entry 4 vs. entry 5). The reduced catalytic activity of compounds containing the ligand in the form of 1,10-phenanthroline is probably due to the increased steric weight of these compounds compared to 2,2′-bipyridyl, which also results in increased steric hindrance that impedes efficient catalysis of isocyanide oligomerization reactions. Correlations between the structure of catalytic activity coincide with the effect of the structure on process efficiency. Moreover, the information gathered through the analysis makes it possible to determine the direction of further research into highly efficient isocyanide oligomerization catalysts. Analysis of the structures proved that O-donor compounds, which provide the greatest stability of the complex, compounds with low steric density, and ligands with high basicity, will perform the best as ligands. The study also provided information about which features of the compounds used as ligands negatively affect the values of catalytic activity and which ligands should not take part in the synthesis of catalysts. Based on this, it can be suggested that further research in this area may focus on using derivatives of the compounds used in the study, e.g., more basic 2,2′-bipyridyl derivatives (e.g., 4,4′-dimethyl-2,2′-bipyridyl,4,4′-bipyridine) or diglycolic acid analogues (e.g., diglycolyl chloride or tetraglycolic acid), which could result in even higher values of catalytic activity with high yields at the same time.

**Figure 7:**
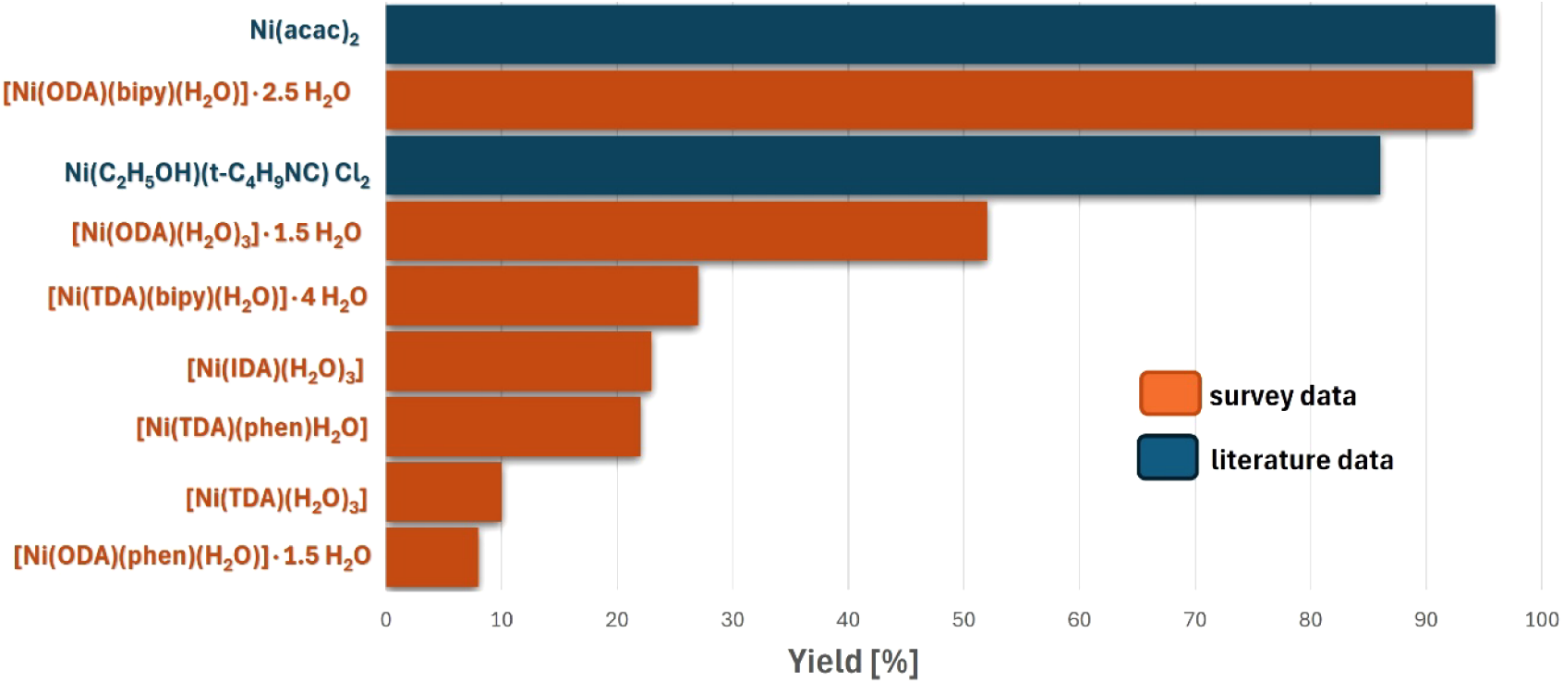
Comparison of performance in the cyclohexyl isocyanide oligomerization reaction of the compounds presented in the study with literature data.^11^

According to data presented in the literature,^11^ the polymerization process of cyclohexyl isocyanide using Ni(acac)_2_ leads to the desired polymer with a yield of 96%. While the use of Ni(C_2_H_5_OH)(t-C_4_H_9_NC)Cl_2_ as a catalyst leads to a product with a yield of 86%. The nickel(II) metal ion complex compounds presented in the study mostly lead to poly(cyclohexyl isocyanide) with low yields (8–52%). In contrast, the [Ni(ODA)(bipy)(H_2_O)]·2.5 H_2_O compound leads to its preparation with yields as high as 94%, which is comparable to or higher than the yields achieved using literature compounds (Figure 7), allowing it to be qualified as a new-generation catalyst to produce poly(cyclohexyl isocyanide).

To date, the literature has not reported a method for calculating catalytic activity in the oligomerization of isocyanides, while the activity of catalytic systems is compared with the yield of the obtained product. However, standard calculations of catalytic activity, in oligomerization processes of other monomers, are based on the mass of oligomer obtained, the duration and conditions of the process, and the amount of catalyst used.

Unfortunately, the use of catalysts based on nickel(II) complex compounds in the polymerization of isocyanides also has its limitations. First of all, the catalytic systems based on nickel(II) ions used in the study belong to the group of heterogeneous catalysts, which can result in low catalytic activity in sterically hindered oligomerization processes of large monomers, and also cause problems in defining the initiation process and controlling the molecular weight of the polymer.^15^ Another limitation in the use of nickel complex compounds is their poor solubility in ethanol, which is the main solvent with which the reaction is conducted. The difficult solubility of nickel compounds requires the use of additional means to accelerate dissolution, for example, an ultrasonic bath, which significantly prolongs the procedure for preparing compounds for oligomerization and may affect the contamination of final products with inaccurately dissolved catalyst residues.

## Conclusion

The presented results show that all of the presented nickel(II) compounds successfully led to the preparation of poly(cyclohexyl isocyanide), with yields ranging from low or moderate (8–52%) to remarkably high up to 94%. In addition, the quantitative and qualitative analyses conducted made it possible to thoroughly examine the obtained products, determine their structure, and confirm their physicochemical properties. The compounds presented in this work represent a group of new catalysts for the oligomerization of cyclohexyl isocyanide not previously described in the literature. Moreover, the compound [Ni(ODA)(bipy)(H_2_O)]·2.5 H_2_O leads to the desired oligomers with yields comparable to the previously used catalysts. Polycarboxylate nickel(II) coordination compounds have not previously been used as catalysts for the oligomerization of isocyanides, and the literature has a dearth of information about the effect of their structure on the activity in this reaction. The presented research not only presents the use of polycarboxylate complexes as new catalysts but also describes in detail the dependence of their structure on the formation of poly(cyclohexyl isocyanide). The presented information results in the enrichment of the existing literature on this subject, addresses a new, previously unexplored topic, and offers a significant expansion of knowledge in the field of the preparation of poly(isocyanide). This provides a basis for further research into the use of nickel(II) metal ion complex compounds in the oligomerization of isocyanides. In addition, the method used to conduct the process is a modified and safer way to obtain poly(isocyanide). The presented oligomerization method leads to functional materials, limiting the release of their toxic and hazardous vapors into the environment while protecting the respiratory tract of the scientist conducting the reactions. The presented research addresses the important, however, forgotten topic in the literature of isocyanide polymerization, which makes it possible to obtain polymeric materials with unusual properties. This study makes it possible to optimize the method and simplify the process of obtaining poly(isocyanide) in a safer way for humans and the environment. This article also shows that working with isocyanides need not be difficult or uncomfortable or cause health problems. Misconceptions about the high toxicity of this group of compounds should not influence the cessation of their development, especially since reactions involving such systems can lead to interesting and useful end products with high yields. Further research in this direction should focus on scaling up the process and using nickel(II) complexes in the polymerization reactions of cyclohexyl isocyanide and applying them to the polymerization of other isocyanides. Overall, this research may lead to the production of many functional materials and intermediates, which are necessary for the development of the fields of medicine, environmental protection, and organic chemistry. Further functionalization and the application of advanced development methods could lead to the production of new pharmaceuticals, drug carriers, and water purification materials and serve as key elements to the modification of many existing synthetic methods. In our opinion, research on the polymerization and oligomerization of isocyanides should be widely developed. The high reactivity of these compounds allows them to be used and tailored to create materials necessary in everyday life. Optimization of this process could lead to the production of poly(isocyanides) with higher yields in a more environmentally friendly and safer manner. Isocyanides deserve greater recognition from chemists, as there is significant potential for further development and innovation in this field.

## Supporting Information Available

Additional experimental details including results of elementary analysis, description of UV–VIS analysis, and description of FT-IR of the obtained complex compound, MALDI-TOF-MS spectra for the individual oligomerization product, DSC curves for the individual oligomerization products, TGA curves for the individual oligomerization products, and photographs of the obtained products (PDF)
