# Supplementary material for: Optimizing Nickel(II) Complex Catalysts for High-Yield Oligomerization of Cyclohexyl Isocyanide
Source: Inorg Chem. 2025 Mar 3;64(10):4844–53. doi: 10.1021/acs.inorgchem.4c04516 (PMC11920947; doi:10.1021/acs.inorgchem.4c04516)
Supplement: Supplementary file 1 — ic4c04516_si_001.pdf [file ic4c04516_si_001.pdf]

Supporting Information

Optimizing nickel(II) complex catalysts for high-yield oligomerization of cyclohexyl isocyanide

Marta Pawlak*[1], Joanna Drzeżdżon[1], Katarzyna N. Jarzembska[2], Dagmara Jacewicz*[1]

[1] Department of Environmental Technology, Faculty of Chemistry, University of Gdansk, Wita Stwosza 63, 80-308 Gdansk, Poland,

[2] Department of Chemistry, University of Warsaw, Żwirki i Wigury 101, 02-089 Warsaw, Poland

e-mail: dagmara.jacewicz@ug.edu.pl, marta.pawlak@phdstud.ug.edu.pl

Table of Contents

1. **Elementary analysis of the obtained complexes** S3
2. **UV-VIS analysis of the obtained complex compounds** S4
3. **FT-IR analysis of the obtained complex compounds** S10
4. **Comparison of experimental and literature data** S13
5. **Powders formed as a result of oligomerization reactions** S17
6. **MALDI-TOF-MS** S18
7. **The differential scanning calorimetry (DSC)** S25
8. **Simultaneous TGA/DSC thermal analysis** S29

1. Elementary analysis of the obtained complexes

Complex compounds	Percent content [%]							
	%C		%H		%S		%N	
	AE	T	AE	T	AE	T	AE	T
[Ni(IDA)(H_2O)$_3$]	20.95	21.08	4.14	3.95	0.00	0.00	5.96	6.14
[Ni(ODA)(H_2O)$_3$]·1.5 H_2O	17.62	17.67	4.85	4.78	0.00	0.00	0.00	0.00
[Ni(TDA)(H_2O)$_3$]	18.01	18.40	3.69	3.87	12.33	12.29	0.00	0.00
[Ni(ODA)(phen)(H_2O)]·1.5 H_2O	45.92	46.20	3.73	4.10	0.00	0.00	6.71	6.73
[Ni(ODA)(bipy)(H_2O)]·2.5 H_2O	43.21	43.93	4.55	4.63	0.00	0.00	6.83	6.83
[Ni(TDA)(phen)H_2O]	46.84	47.10	3.20	3.30	7.12	7.30	6.90	6.91
[Ni(TDA)(bipy)(H_2O)]·4 H_2O	38.48	38.65	4.46	4.60	7.19	7.36	6.20	6.18

AE- elemental analysis *T*-Theoretical calculations.

Table S1 Elemental analysis results for the synthesized nickel(II) complex compounds.

2. UV-VIS analysis of the obtained complex compounds

Description of UV-VIS spectra

Unfortunately, the substrates used in the synthesis have very similar absorbance ranges, so that the bands largely overlap and there is a problem in distinguishing them for individual ligands. The bands with the highest intensity on the spectra of [Ni(ODA)(bipy)(H_2O)]·2.5 H_2O, [Ni(ODA)(phen)(H_2O)]·1.5 H_2O, [Ni(TDA)(bipy)(H_2O)]·4 H_2O, [Ni(TDA)(phen)H_2O] appear in the 450-200 nm range , these bands come from the ligands 1,10-phenanthroline and 2,2'-bipyridyl. On the spectra of the compounds [Ni(TDA)(H_2O)$_3$], [Ni(ODA)(H_2O)$_3$]·1.5 H_2O and [Ni(IDA)(H_2O)$_3$] the bands with the highest intensity appear in the 300-200 nm range originate from the ligands diglycolic acid, iminodiacetic acid and thiodiacetic acid. Bands of slightly lower intensity are noticeable at 700-480 nm on the spectra of Ni(ODA)(bipy)(H_2O)]·2.5 H_2O, [Ni(ODA)(phen)(H_2O)]·1.5 H_2O, [Ni(TDA)(bipy)(H_2O)]·4 H_2O and [Ni(TDA)(phen)H_2O] and 800-550 nm on the spectra of [Ni(TDA)(H_2O)$_3$], [Ni(ODA)(H_2O)$_3$]· 1.5 H_2O and [Ni(IDA)(H_2O)$_3$]. Comparing this with the $NiCl_2$·6 H_2O spectrum shown, it can be concluded that these are bands originating from the central atom of Ni(II).

UV-VIS spectra of aqueous solutions are comparable to those taken for solids, and the spectra obtained also confirm the structure, the electron transitions that occur, and confirm the formation of complex compounds (**Figure S1-S5**).

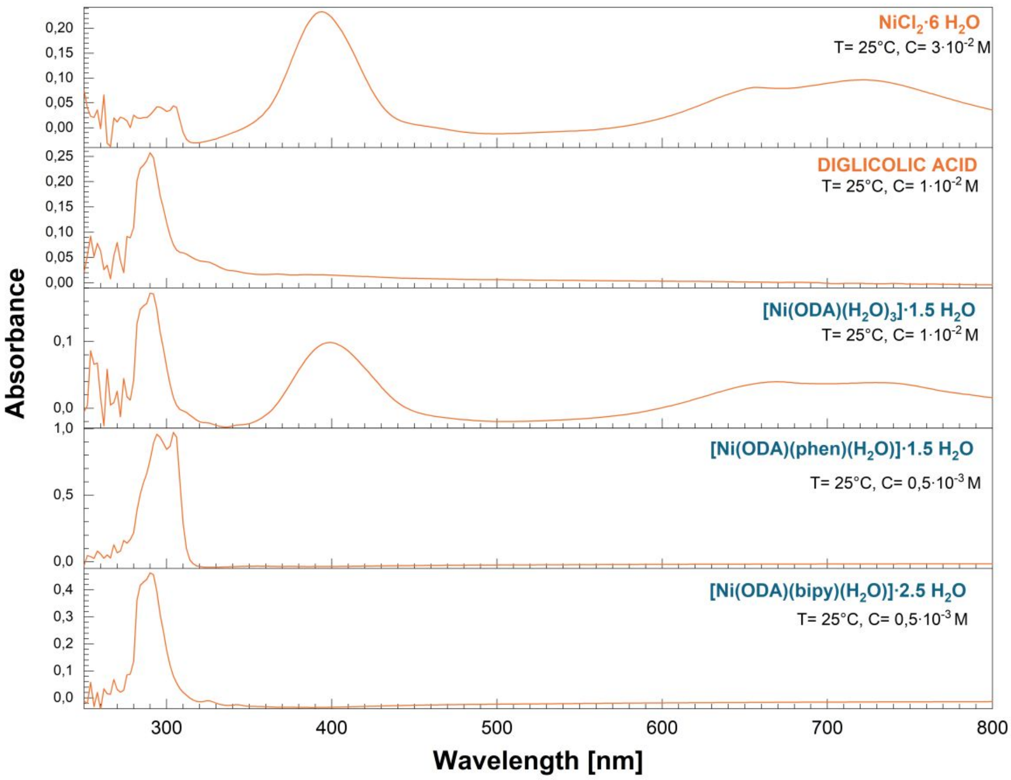

Figure S1 Comparison of aqueous solution UV-VIS spectra of complex compounds with ligand-diglycolate

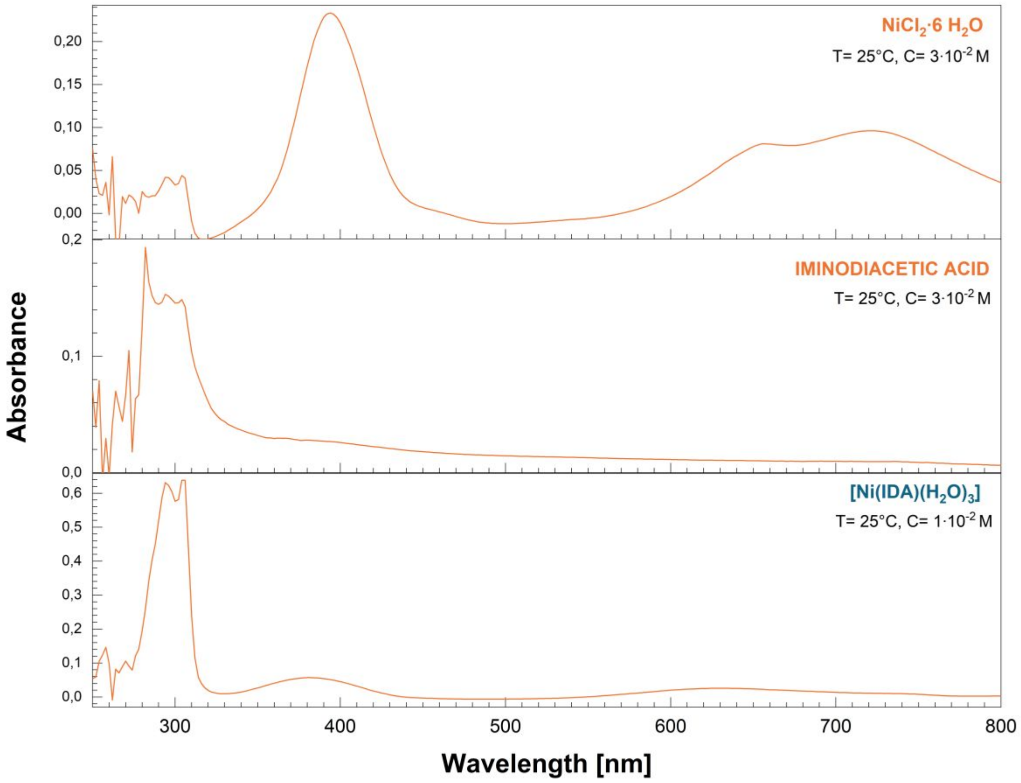

Figure S2 Comparison of aqueous solution UV-VIS spectra of complex compounds with ligand-iminodiacetate

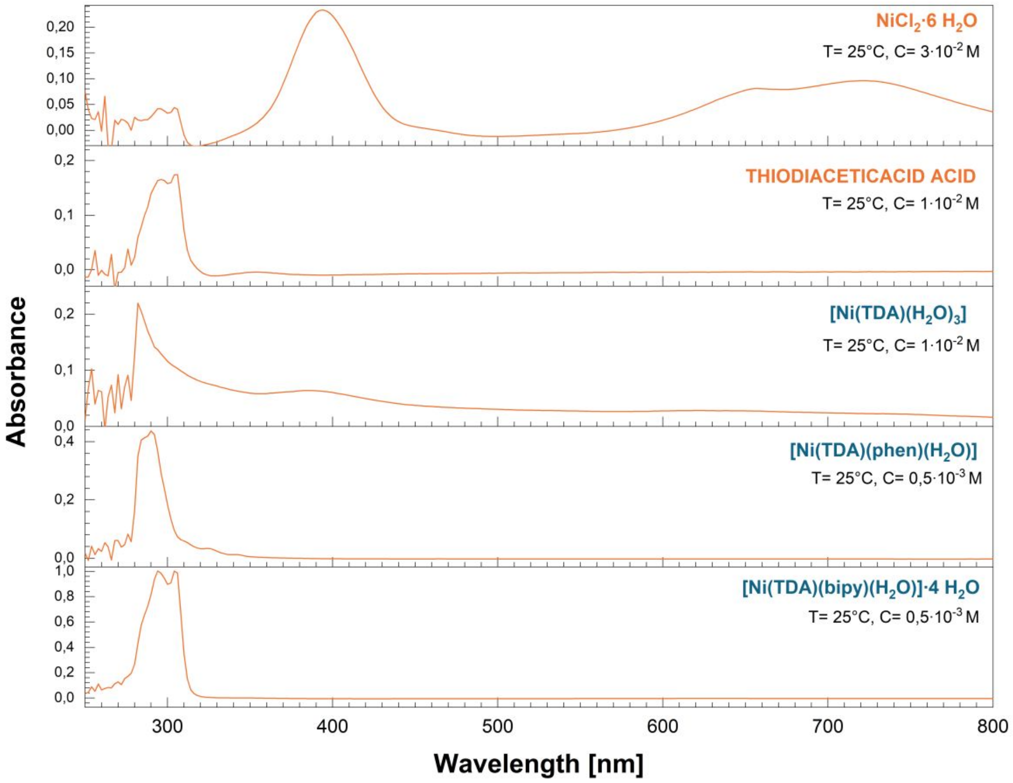

Figure S3 Comparison of aqueous solution UV-VIS spectra of complex compounds with ligand-thiodiacetate

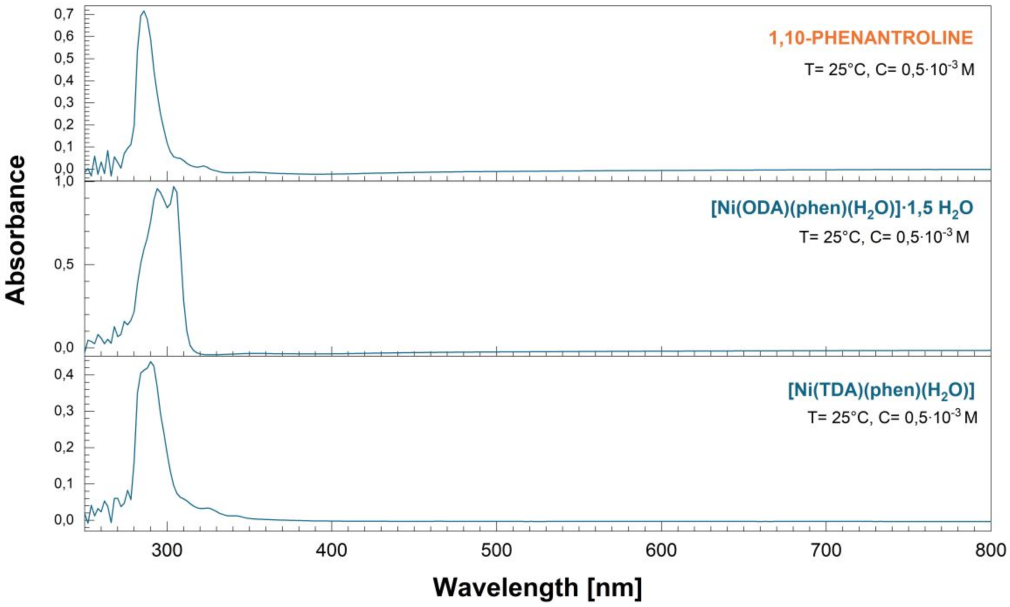

Figure S4 Comparison of aqueous solution UV-VIS spectra of complex compounds with ligand- 1,10-phenantroline

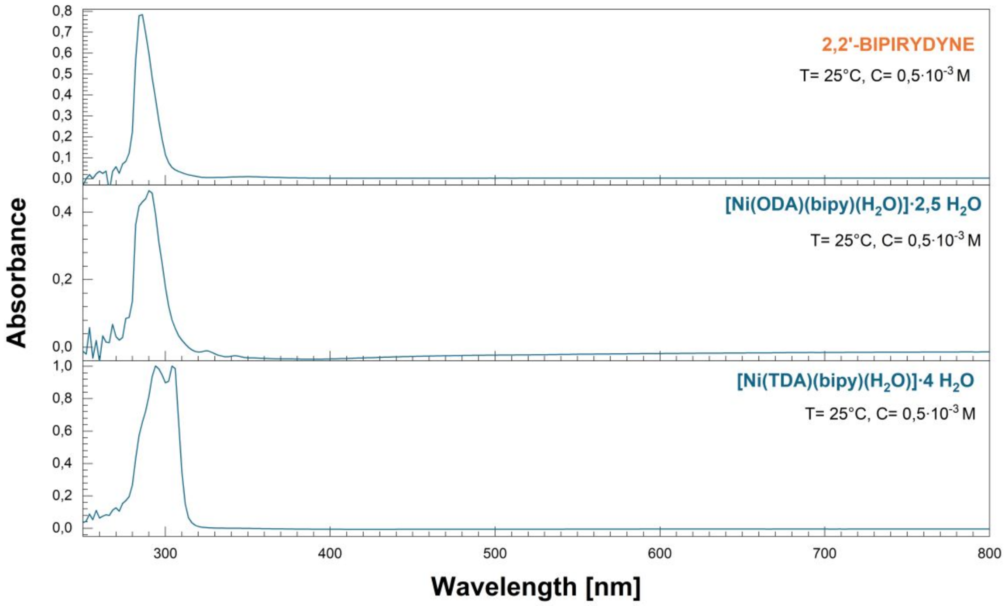

Figure S5 Comparison of aqueous solution UV-VIS spectra of complex compounds with ligand- 2,2'-bipirydyne

3. FT-IR analysis of the obtained complex compounds

Each of the obtained complex compounds was subjected to FT-IR analysis to confirm their structure. FT-IR spectra for all complexes were tabulated and compared (**Figure S6**), and the resulting bands are summarized and described in **Table S2**.

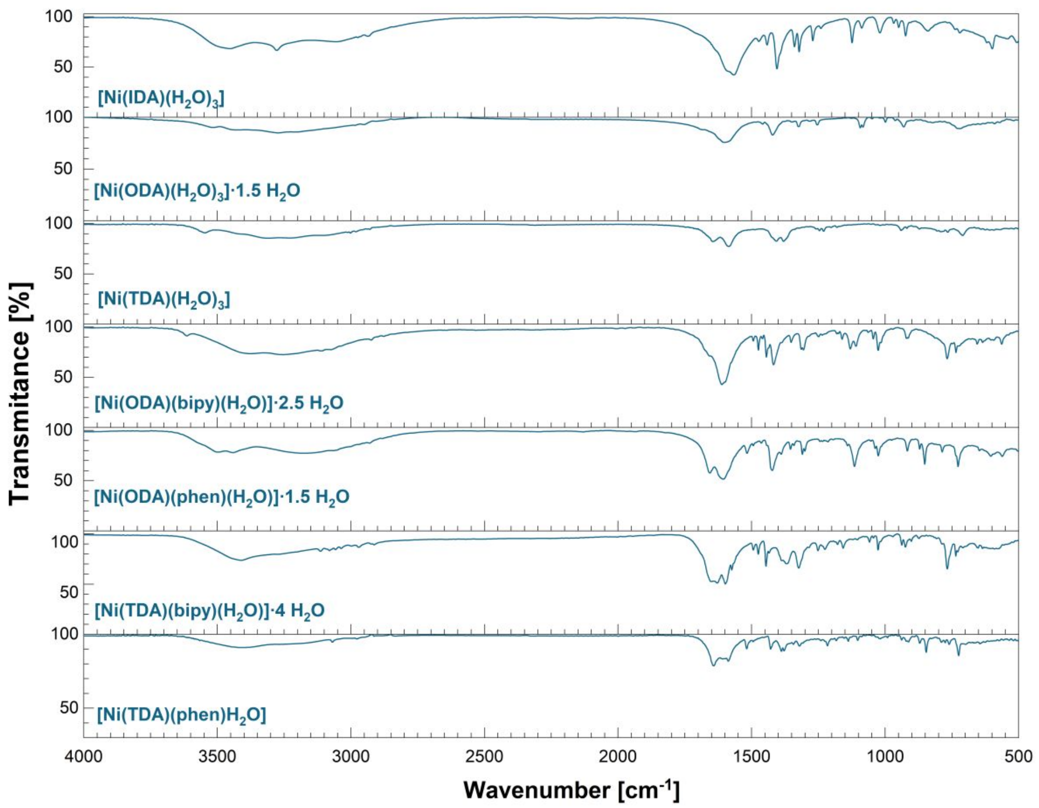

Figure S6. Comparison of the FTIR spectra of the compounds used during the study to identify characteristic functional groups and confirm compound structures

Table S2. Summary of bands present on FT-IR spectra of analyzed complex compounds

WAVENUMBER [cm^{-1}]	STRUCTURAL ELEMENT OBSERVED	STRUCTURAL ELEMENT ORIGIN	COMPLEX COMPOUND
3500-3300	-OH	water molecule in the structure	[Ni(IDA)(H_2O)$_3$]<br>[Ni(ODA)(H_2O)$_3$]·1.5 H_2O<br>[Ni(TDA)(H_2O)$_3$]

			$[Ni(ODA)(bipy)(H_2O)]\cdot 2.5\ H_2O$<br>$[Ni(ODA)(phen)(H_2O)]\cdot 1.5\ H_2O$<br>$[Ni(TDA)(bipy)(H_2O)]\cdot 4\ H_2O$<br>$[Ni(TDA)(phen)H_2O]$
3100<br>1500<br>1000<br>500	pyridine ring	ring present in the ligand structure	$[Ni(ODA)(bipy)(H_2O)]\cdot 2.5\ H_2O$<br>$[Ni(ODA)(phen)(H_2O)]\cdot 1.5\ H_2O$<br>$[Ni(TDA)(bipy)(H_2O)]\cdot 4\ H_2O$<br>$[Ni(TDA)(phen)H_2O]$
1650-1580<br>1300-1200	R-NH-CH_2-R	group present in the iminodiacetic acid ligand stucture	$[Ni(IDA)(H_2O)_3]$
1270<br>1150	R-O-CH-R	group present in the diglicolic acid ligand stucture	$[Ni(ODA)(H_2O)_3]\cdot 1.5\ H_2O$<br>$[Ni(ODA)(bipy)(H_2O)]\cdot 2.5\ H_2O$<br>$[Ni(ODA)(phen)(H_2O)]\cdot 1.5\ H_2O$
1720-1700	R-CH=O	group present in the stucture of acid ligands	$[Ni(IDA)(H_2O)_3]$<br>$[Ni(ODA)(H_2O)_3]\cdot 1.5\ H_2O$<br>$[Ni(TDA)(H_2O)_3]$<br>$[Ni(ODA)(bipy)(H_2O)]\cdot 2.5\ H_2O$<br>$[Ni(ODA)(phen)(H_2O)]\cdot 1.5\ H_2O$

			$[Ni(TDA)(bipy)(H_2O)]\cdot 4\ H_2O$<br>$[Ni(TDA)(phen)H_2O]$
1610-1540	R-O-CH=O	group present in the stucture of acid ligands	$[Ni(IDA)(H_2O)_3]$<br>$[Ni(ODA)(H_2O)_3]\cdot\ 1.5\ H_2O$<br>$[Ni(TDA)(H_2O)_3]$<br>$[Ni(ODA)(bipy)(H_2O)]\cdot 2.5\ H_2O$<br>$[Ni(ODA)(phen)(H_2O)]\cdot 1.5\ H_2O$<br>$[Ni(TDA)(bipy)(H_2O)]\cdot 4\ H_2O$<br>$[Ni(TDA)(phen)H_2O]$
800-700	Ni-N	bonding of the ligand to the metallic center	$[Ni(IDA)(H_2O)_3]$
800-700	Ni-S	bonding of the ligand to the metallic center	$[Ni(TDA)(H_2O)_3]$<br>$[Ni(TDA)(bipy)(H_2O)]\cdot 4\ H_2O$<br>$[Ni(TDA)(phen)H_2O]$
800-700	Ni-O	bonding of the ligand to the metallic center	$[Ni(ODA)(H_2O)_3]\cdot 1.5\ H_2O$<br>$[Ni(ODA)(bipy)(H_2O)]\cdot 2.5\ H_2O$<br>$[Ni(ODA)(phen)(H_2O)]\cdot 1.5\ H_2O$

4. Comparison of experimental and literature data

Table S3 Comparison table of elemental analysis results and spectroscopic data obtained during the exercise and data from the reference literature.

	EXPERIMENTAL DATA			LITERATURE DATA		
	UV-VIS	IR ANALYSIS	ELEMENTAR ANALYSIS	UV-VIS	IR ANALYSIS	REF.
**[Ni(IDA)(H_2O)$_3$]**	λ_{max} 620 nm 390 nm	3500-3300 cm^{-1} (ν OH), 1720-1700 cm^{-1} (ν_{as} COO^-) 1610-1540 cm^{-1} (ν_{as} COO^-) 1400-1300 cm^{-1} ($\nu_{sym}COO^-$) 1300-1200 cm^{-1} (ν_{as} CNH) 800-700 ($\nu_{sym}Ni$-N)	C, 20,9%; H, 4,14%; N, 5,96%	-	-	-
**[Ni(ODA)(H_2O)$_3$]·1.5 H_2O**	λ_{max} 620 nm 395 nm 235 nm	350-3300 cm^{-1} ($\nu_{as}OH$), 1720-1700 cm^{-1} (ν_{as} COO^-) 1610-1540 cm^{-1} (ν_{as} COO^-) 1400-1300 cm^{-1} ($\nu_{sym}COO^-$) 1270 cm^{-1} 1150 cm^{-1} ($\nu_{as}OCH$) 800-600 cm^{-1} ($\nu_{sym}Ni$-O)	C, 17,6%; H, 4.85%;	-	1607 cm^{-1},1458 cm^{-1} 1422 cm^{-1},1348 cm^{-1} 1327 cm^{-1},1281 cm^{-1} 1255 cm^{-1},1094 cm^{-1} 1084 cm^{-1},1049 cm^{-1} 999 cm^{-1}, 962 cm^{-1} 932 cm^{-1},723 cm^{-1} 590 cm^{-1}, 527 cm^{-1} 374 cm^{-1}	[21,23]

**[Ni(TDA)(H_2O)$_3$]**	λ_{max} 650 nm 405 nm 290 nm	3550-2900 cm^{-1} ($\nu\, OH$), 1720-1700 cm^{-1} (ν_{as} COO^{-}) 1610-1540 cm^{-1} (ν_{as} COO^{-}) 1400-1350 cm^{-1} (ν_{sym} COO^{-}) 800-700 cm^{-1} ($\nu_{sym}Ni$ -S)	C, 18.1%; H, 3.7%; S, 12.3%	-	3547 cm^{-1} , 3313 cm^{-1}, 3212 cm^{-1} ($\nu_{as}TDA$) 1642 cm^{-1} ($\nu_{sym}TDA$) 2981 cm^{-1} ($\nu_{as}CH_2$) 2928 cm^{-1} ($\nu_{sym}CH_2$) 1590 cm^{-1} ($\nu_{as}COO^{-}$) 1382 cm^{-1} ,1373 cm^{-1} ($\nu_{sym}COO^{-}$) 713 cm^{-1} (ν_{sym}C-S)	[19]
**[Ni(ODA)(phen)(H_2O)]·1.5 H_2O**	λ_{max} 610 nm 365 nm 265 nm	3500-3300 cm^{-1} ($\nu\, OH$), 3100 cm^{-1} (ν pyridine ring) 1720-1700 cm^{-1} (ν_{as} COO^{-}) 1610-1540 cm^{-1} (ν_{as} COO^{-}), 1500 cm^{-1} ($\nu_{sym}COO^{-}$), (ν pyridine ring) 1400-1300 cm^{-1} (ν_{sym} COO^{-}), 1270 cm^{-1},1150 cm^{-1}, ($\nu_{as}OCH$) 1000 cm^{-1} (ν pyridine ring), 800-700 cm^{-1} ($\nu_{as}Ni$-O) 600-500 cm^{-1}	C, 45,9%; H, 3.7%; N, 6,7%		1657 cm^{-1} ,1607 cm^{-1} ($\nu_{as}COO^{-}$) 1518 cm^{-1} ($\nu\, phen$) 1495 cm^{-1} ,1424 cm^{-1} ($\nu_{sym}COO^{-}$) 1388 cm^{-1},1354 cm^{-1}, 1310 cm^{-1},1300 cm^{-1} 1115 cm^{-1},1026 cm^{-1}, 918 cm^{-1},852 cm^{-1} 787 cm^{-1}, 727 cm^{-1} ($\nu\, phen$) 604 cm^{-1}, 563 cm^{-1}, 258 cm^{-1}	[21,23]

**[Ni(ODA)(bipy)(H_2O)]·2.5 H_2O**	λ_{max} 615 nm 360 nm 305 nm	3500-3300 cm^{-1} (ν OH), 3100 cm^{-1} (ν pyridine ring) 1720-1700 cm^{-1} (ν_{as} COO^{-}) 1610-1540 cm^{-1} (ν_{as} COO^{-}), 1500 cm^{-1} (ν pyridine ring) 1400-1300 cm^{-1} (ν_{sym} COO^{-}), 1270 cm^{-1},1150 cm^{-1}, ($\nu_{as}OCH$) 1000 cm^{-1} (ν pyridine ring) 800-600 cm^{-1} ($\nu_{as}Ni$-O) 600-500 cm^{-1}	C, 43.2%; H, 4.55%; N, 6.83%	-	3625 cm^{-1}, 3400-3000 cm^{-1},1660 cm^{-1}, 1605 cm^{-1},1450 cm^{-1}, 1426 cm^{-1},1385 cm^{-1}, 1357 cm^{-1},1311 cm^{-1}, 1256 cm^{-1},1243 cm^{-1}, 1187 cm^{-1},1164 cm^{-1}, 1151 cm^{-1},1073 cm^{-1}, 1050 cm^{-1},1041 cm^{-1}, 1027 cm^{-1},1013 cm^{-1}, 981 cm^{-1}, 930 cm^{-1} 907 cm^{-1}, 774 cm^{-1} 742 cm^{-1},728 cm^{-1} 660 cm^{-1},637 cm^{-1}	[21,23]
**[Ni(TDA)(phen)H_2O]**	λ_{max} 590 nm 360 nm 260 nm	3500-3300 cm^{-1} (ν_{as} OH), 3100 cm^{-1} (ν pyridine ring) 1720-1700 cm^{-1} (ν_{as} COO^{-}) 1610-1540 cm^{-1} (ν_{as} COO^{-}), 1500 cm^{-1} ($\nu_{sym}COO^{-}$), (ν pyridine ring) 1400-1300 cm^{-1} (ν_{sym} COO^{-})	C, 46.8%; H, 3.2%; N, 7,12%; S, 6,9 %	λ_{max} 255 nm 365 nm 585 nm	1586 cm^{-1}($\nu_{as}COO^{-}$) 1600 cm^{-1}($\nu_{sym}COO^{-}$)	[20]

		1000 cm^{-1} (ν pyridine ring)<br>800-700 cm^{-1} (ν_{as} $Ni-S$)<br>500 cm^{-1}($\nu_{sym}C$-S)				
**[Ni(TDA)(bipy)(H_2O)]·4 H_2O**	λ_{max}<br>590 nm<br>295 nm	3500-3300 cm^{-1} (ν_{as} OH),<br>3100 cm^{-1}<br>(ν pyridine ring)<br>1720-1550 cm^{-1} (ν_{as} COO^{-})<br>1610-1540 cm^{-1}<br>($\nu_{as}COO^{-}$),<br>1500 cm^{-1}($\nu_{as}COO^{-}$),<br>(ν pyridine ring)<br>1400-1300 cm^{-1} (ν_{sym} COO^{-})<br>1000 cm^{-1} (ν pyridine ring)<br>800-700 cm^{-1} ($\nu_{as}Ni$ $-S$)<br>500 cm^{-1}($\nu_{sym}C$-S)	C, 38,46%;<br>H, 4,46%;<br>N, 7.2%;<br>S, 6,2%	λ_{max}<br>300 nm<br>585 nm	1620 cm^{-1} ($\nu_{as}COO^{-}$<br>1384 cm^{-1} ($\nu_{sym}COO^{-}$)	[20]

5. Powders formed as a result of oligomerization reactions

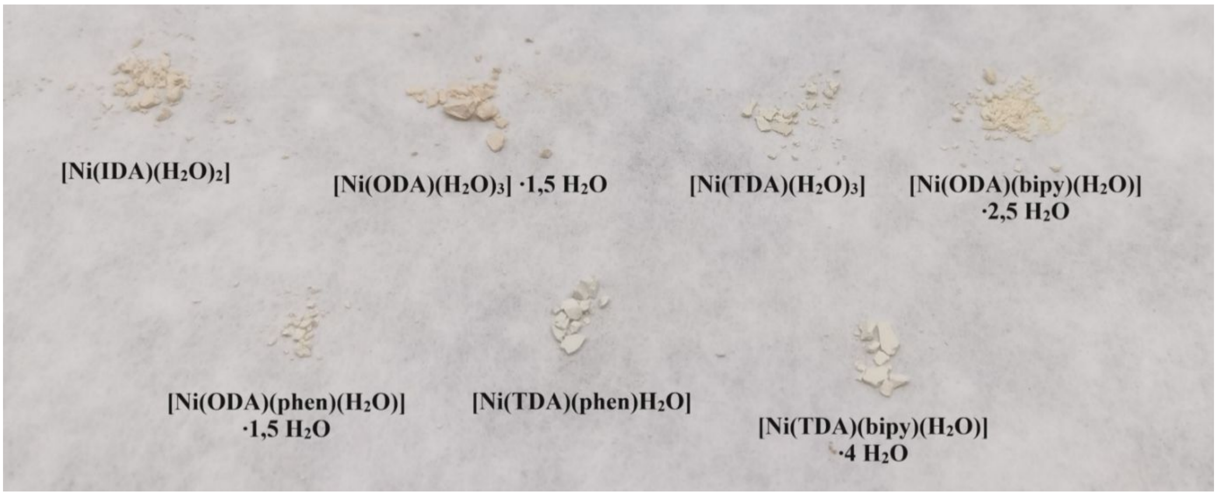

Figure S7 Comparison of powders formed by oligomerization reactions.

6. MALDI-TOF-MS

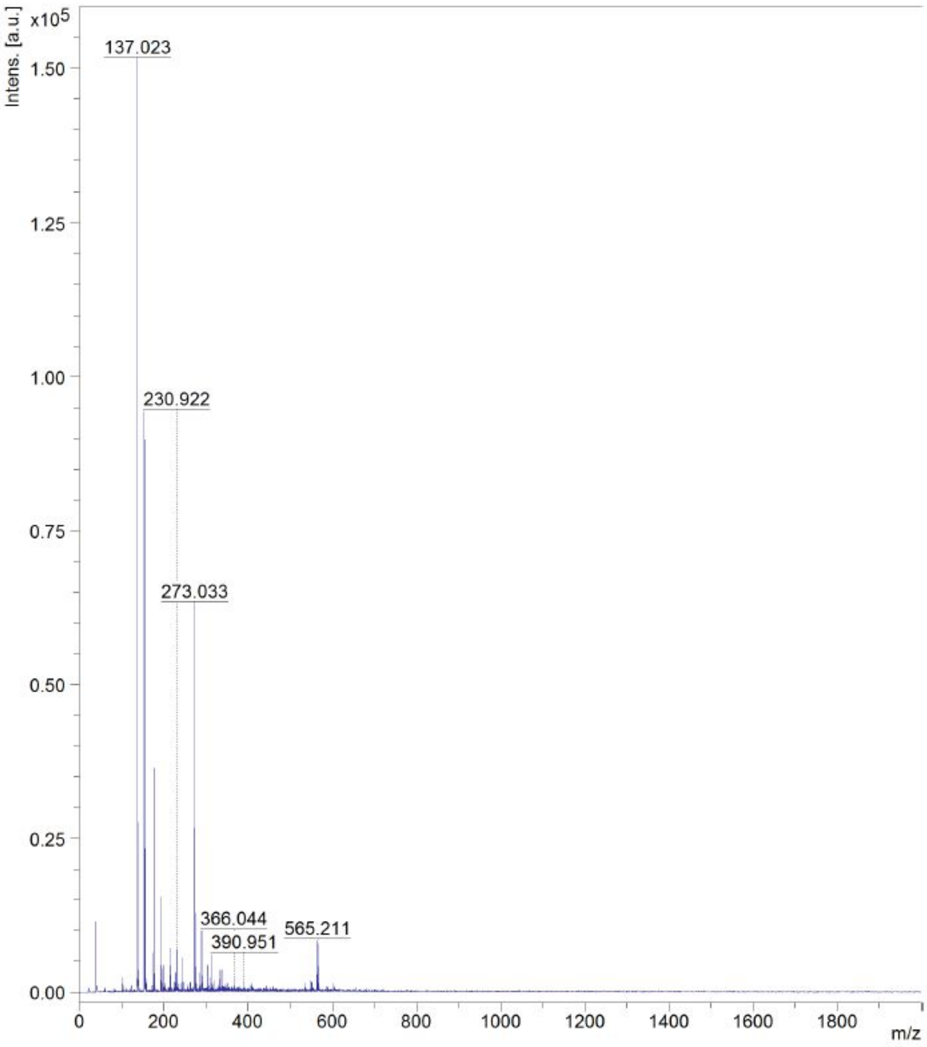

Figure S8 MALDI-TOF-MS spectrum of the cyclohexyl isocyanide the oligomerization product catalyzed by the [Ni(IDA)(H_2O)$_2$]

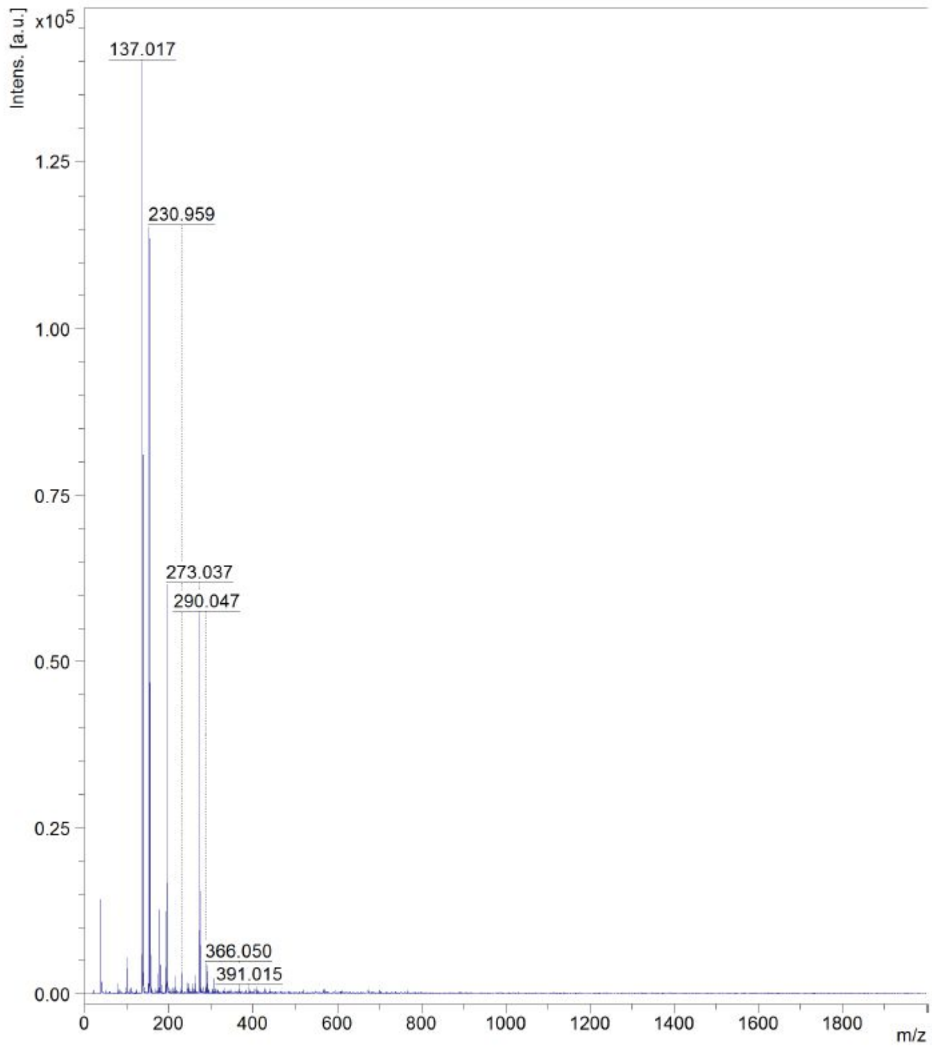

Figure S9 MALDI-TOF-MS spectrum of the cyclohexyl isocyanide the oligomerization product catalyzed by the [Ni(ODA)(H_2O)$_3$]·1.5 H_2O

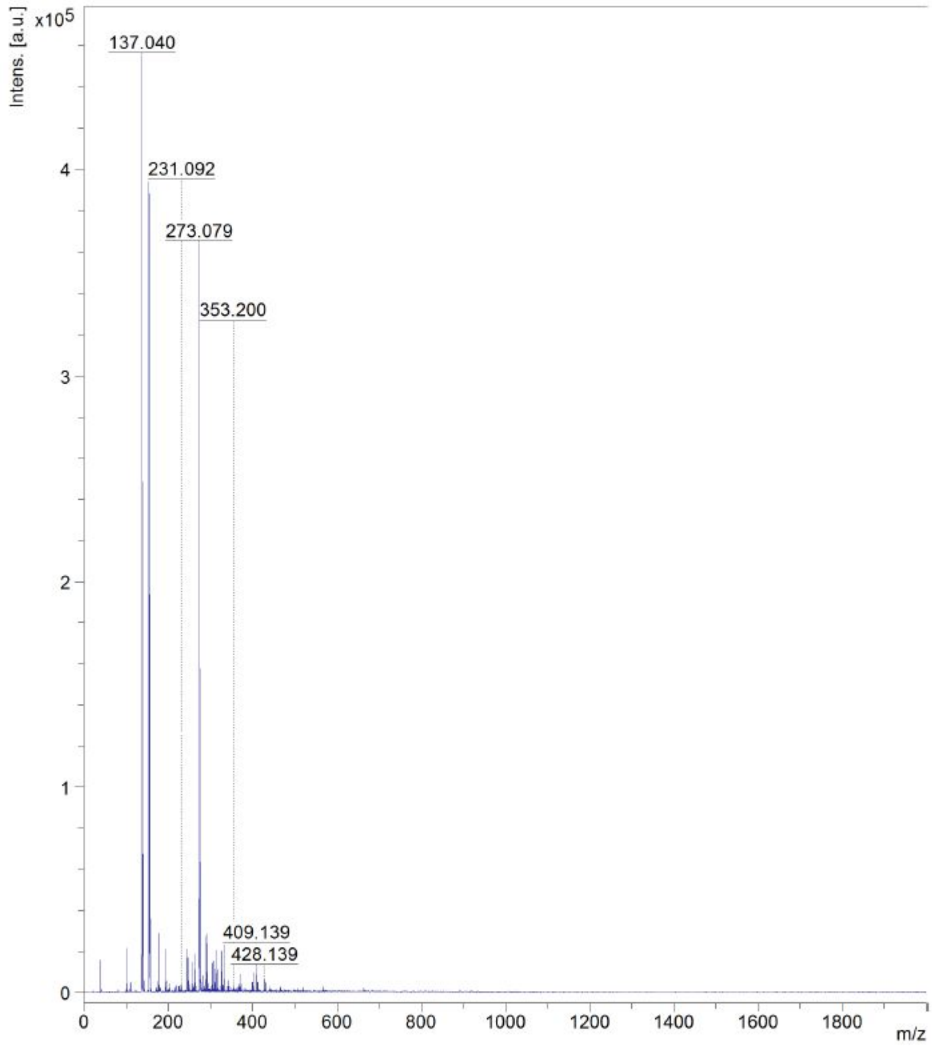

Figure S10 MALDI-TOF-MS spectrum of the cyclohexyl isocyanide the oligomerization product catalyzed by the [Ni(TDA)(H_2O)$_3$]

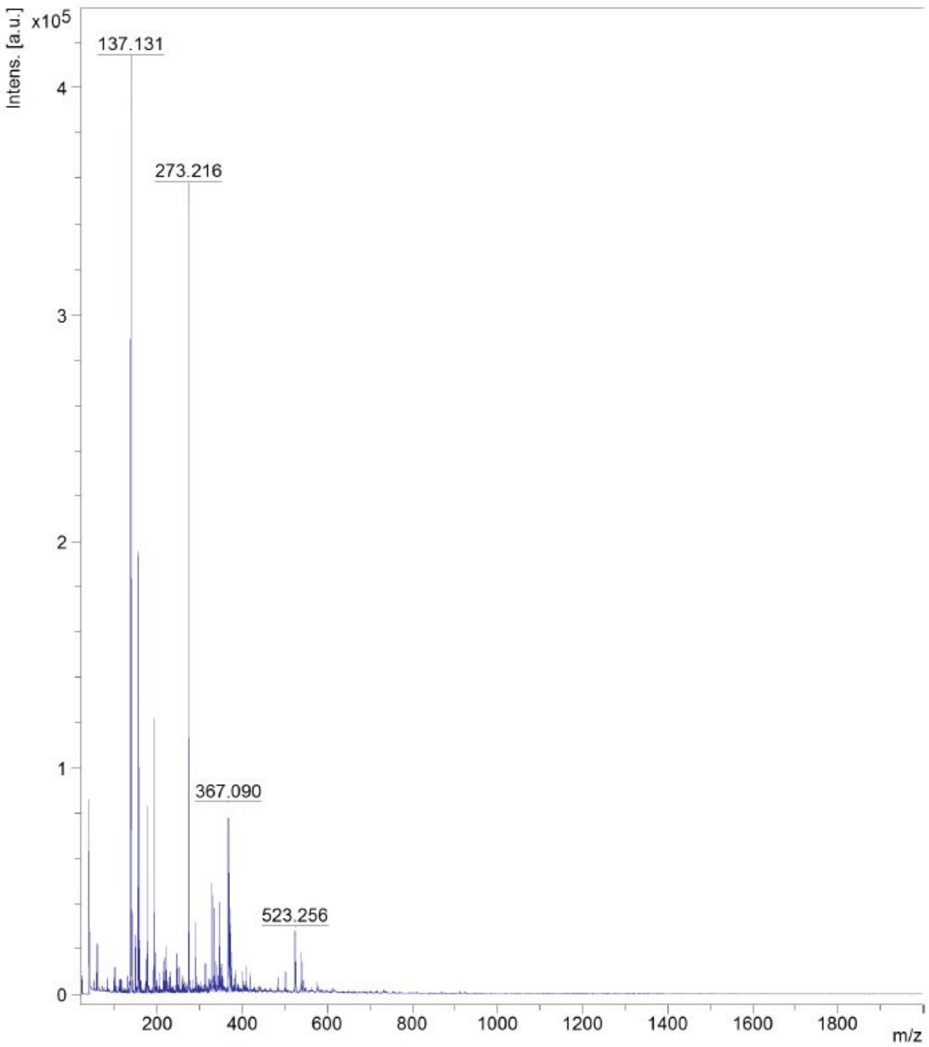

Figure S11 MALDI-TOF-MS spectrum of the cyclohexyl isocyanide the oligomerization product catalyzed by the [Ni(ODA)(bipy)(H_2O)]·2.5 H_2O

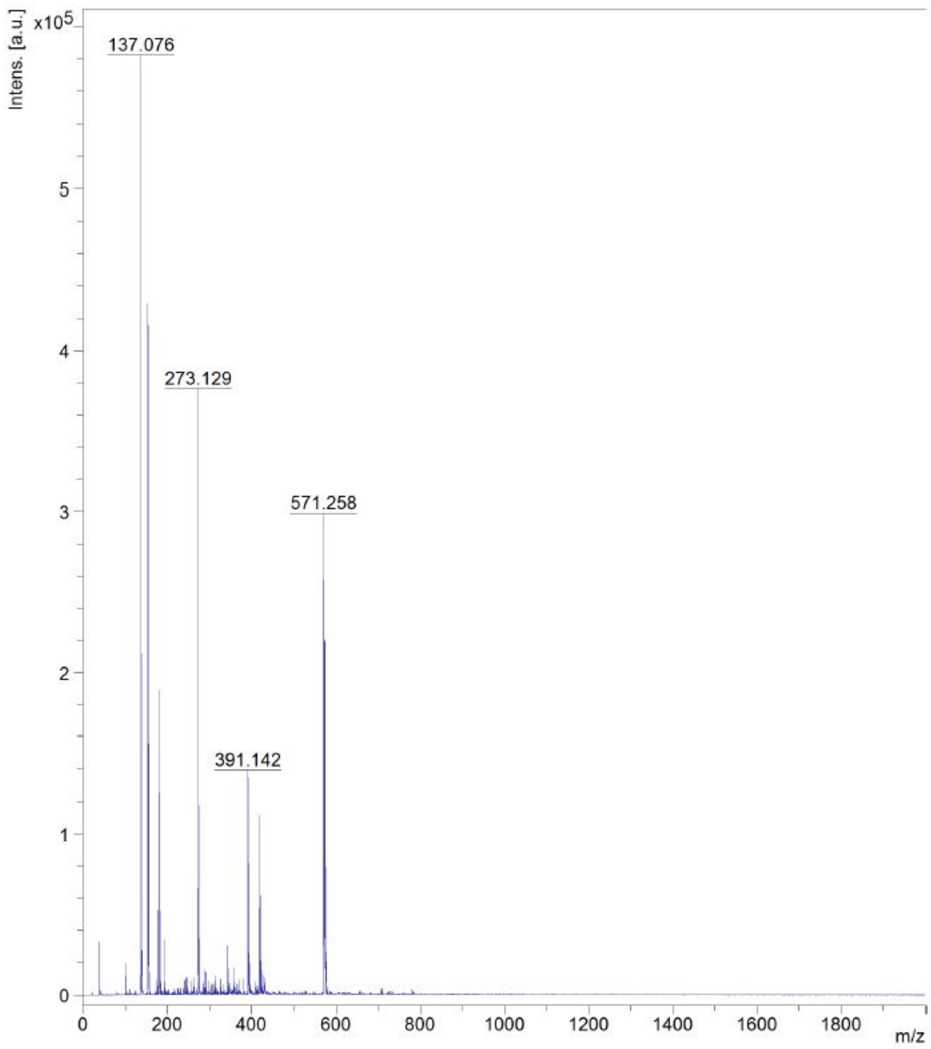

Figure S12 MALDI-TOF-MS spectrum of the cyclohexyl isocyanide the oligomerization product catalyzed by the [Ni(ODA)(phen)(H_2O)]·1.5 H_2O

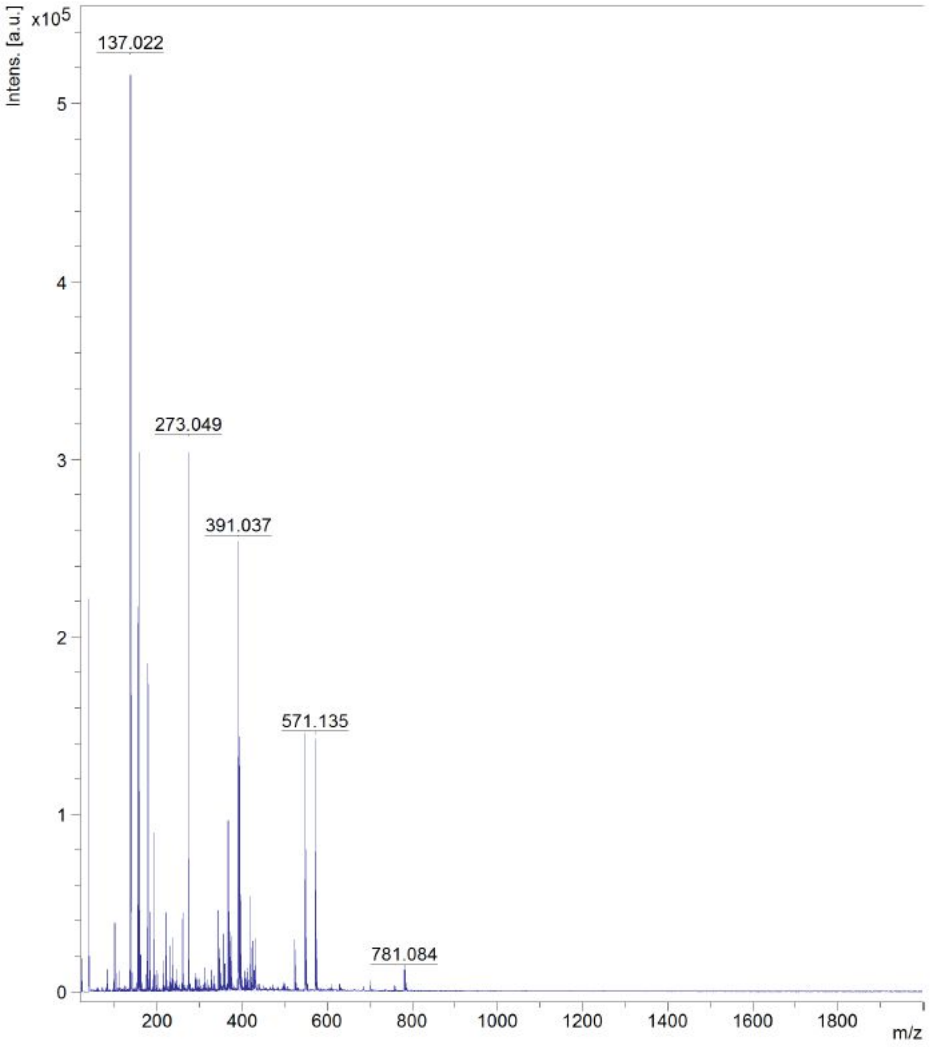

Figure S13 MALDI-TOF-MS spectrum of the cyclohexyl isocyanide the oligomerization product catalyzed by the [Ni(TDA)(bipy)(H_2O)]·4 H_2O

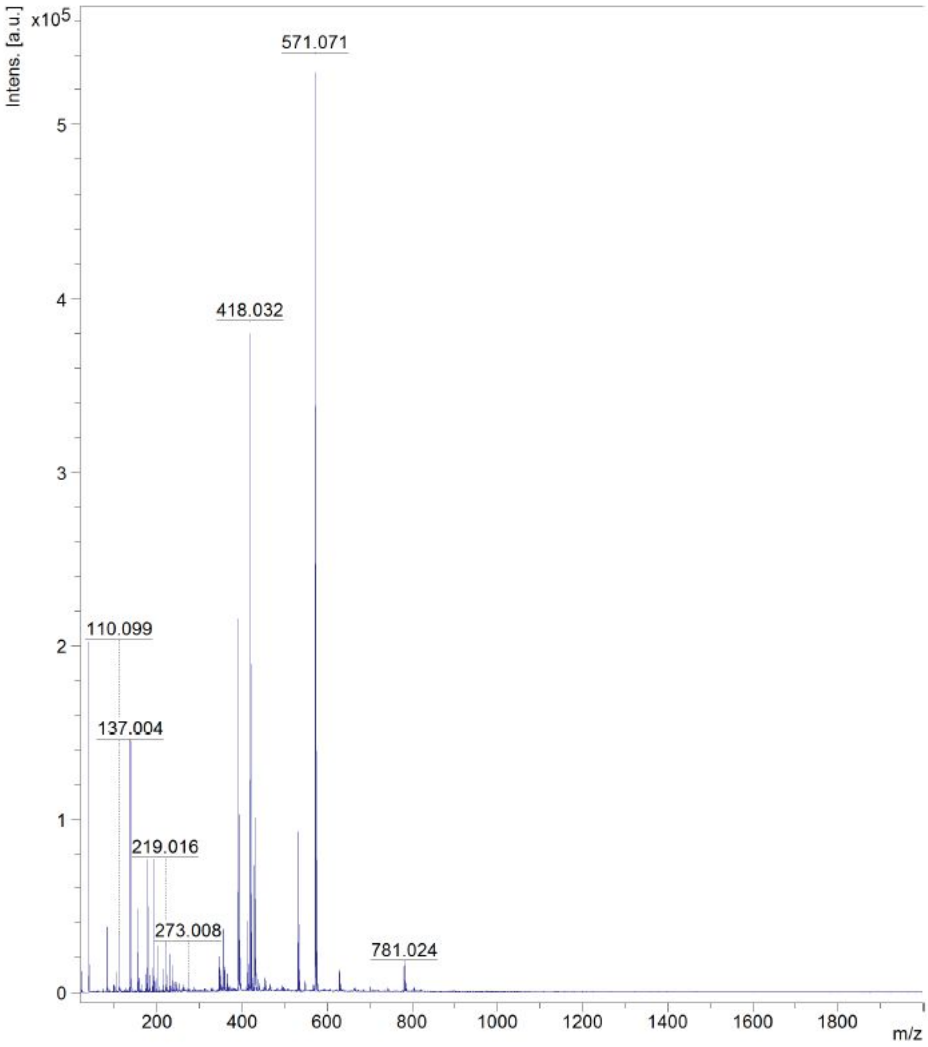

Figure S14 MALDI-TOF-MS spectrum of the cyclohexyl isocyanide the oligomerization product catalyzed by the [Ni(TDA)(phen)H_2O]

7. The differential scanning calorimetry (DSC)

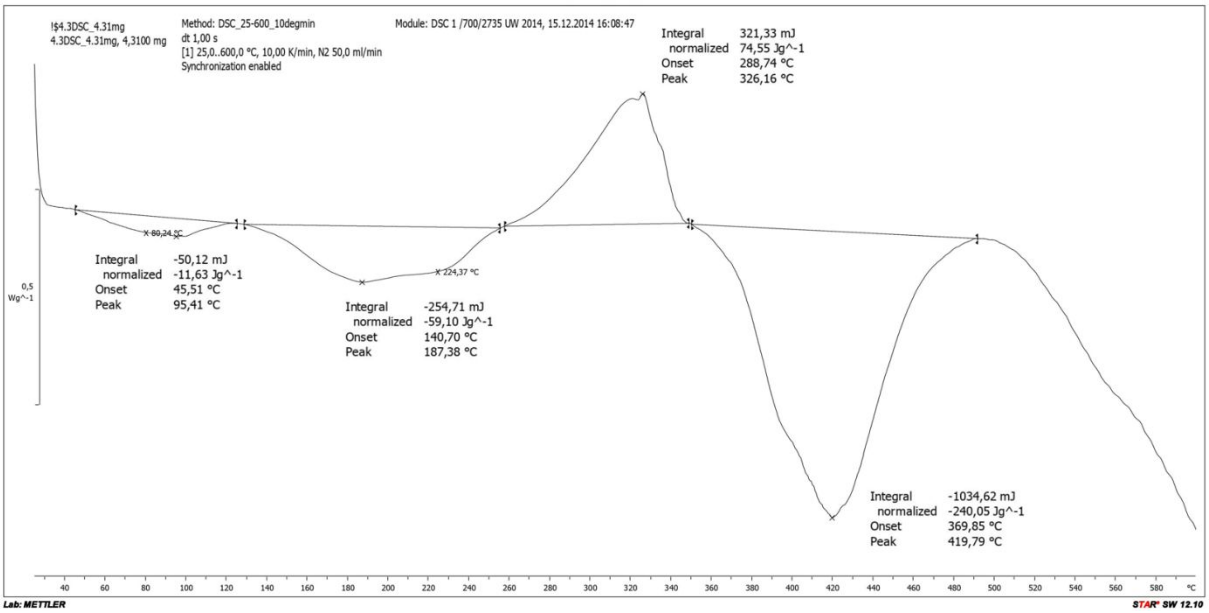

Figure S15 DSC curve of the cyclohexyl isocyanide the oligomerization product catalyzed by the [Ni(IDA)(H_2O)$_2$]

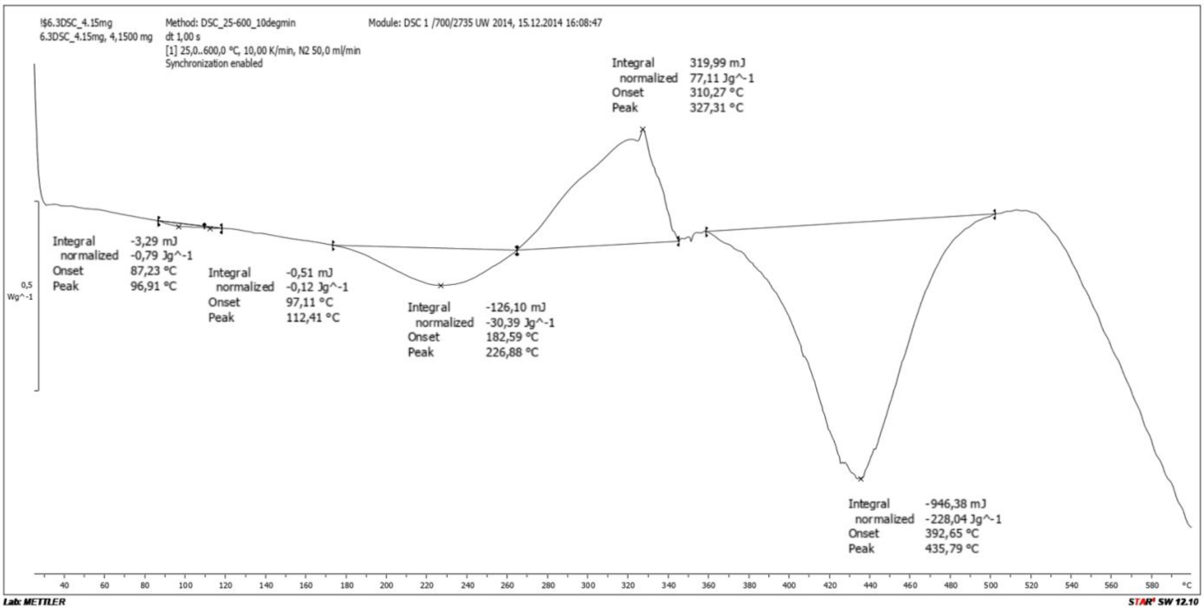

Figure S16 DSC curve of the cyclohexyl isocyanide the oligomerization product catalyzed by the [Ni(ODA)(H_2O)$_3$]·1.5 H_2O

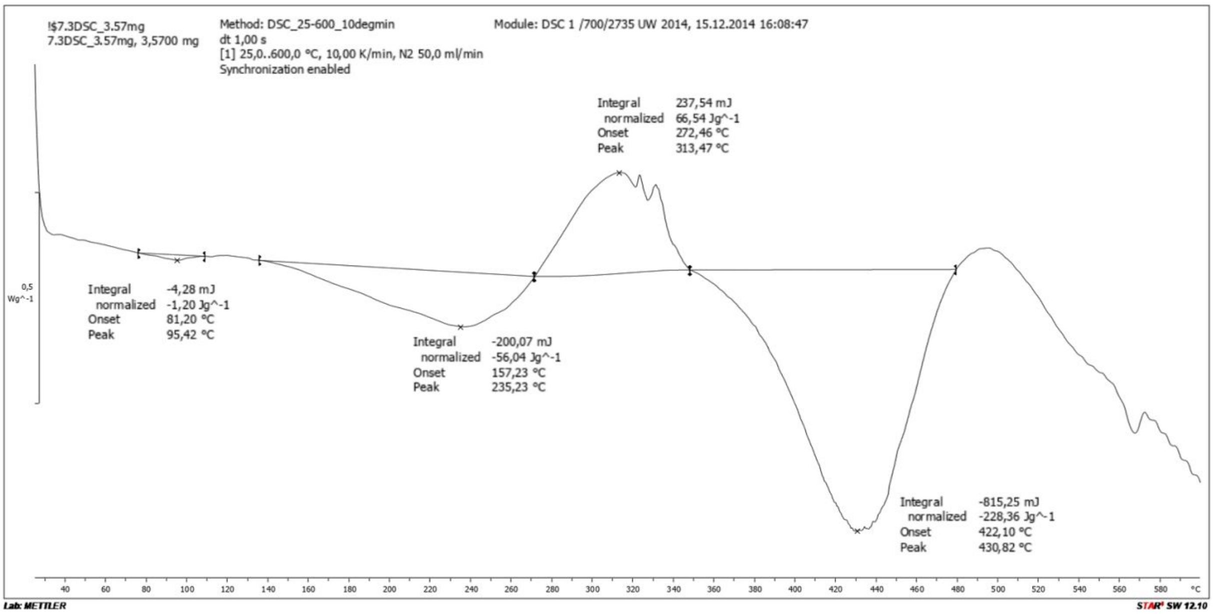

Figure S17 DSC curve of the cyclohexyl isocyanide the oligomerization product catalyzed by the [Ni(TDA)(H_2O)$_3$]

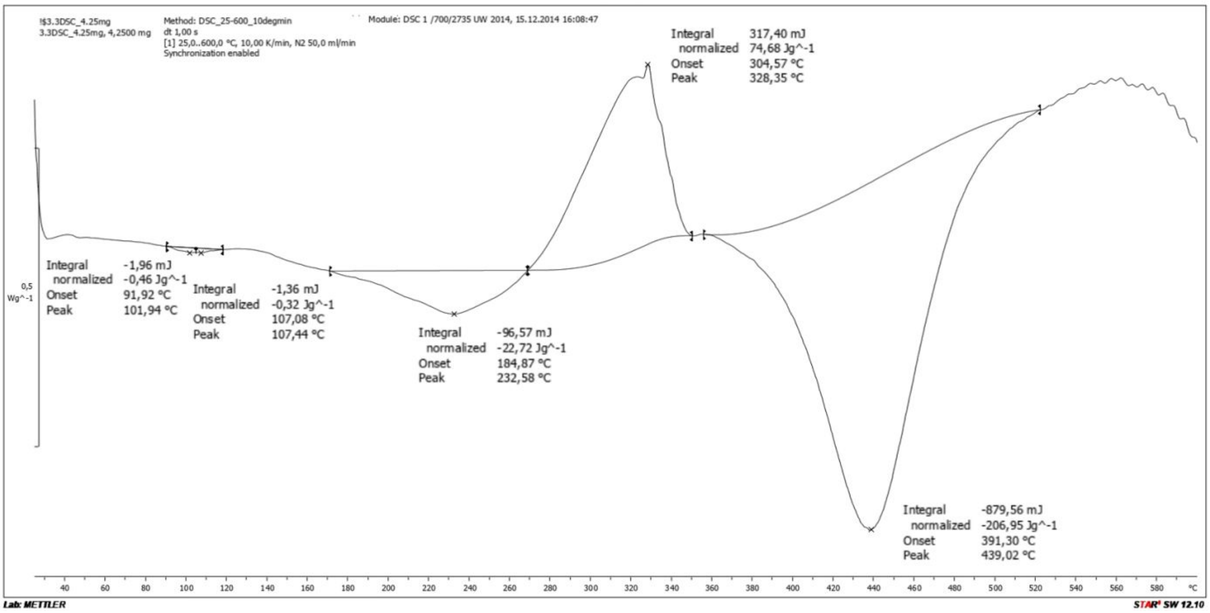

Figure S18 DSC curve of the cyclohexyl isocyanide the oligomerization product catalyzed by the [Ni(ODA)(phen)(H_2O)]·1.5 H_2O

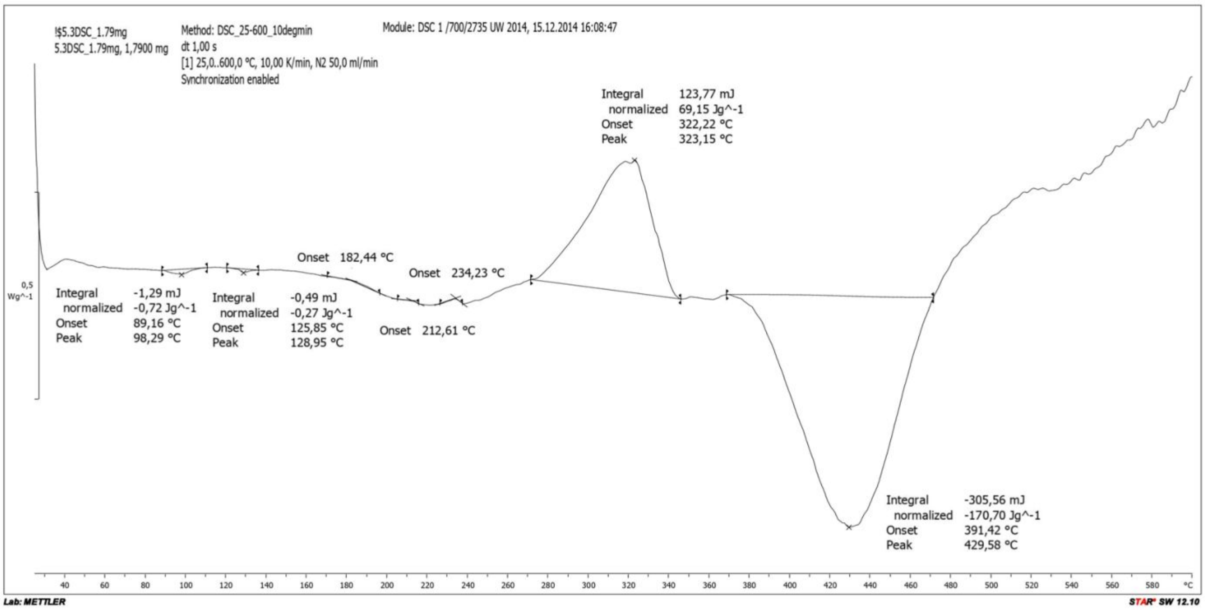

Figure S19 DSC curve of cyclohexyl the isocyanide the oligomerization product catalyzed by the $[Ni(ODA)(bipy)(H_2O)]\cdot 2.5\ H_2O$

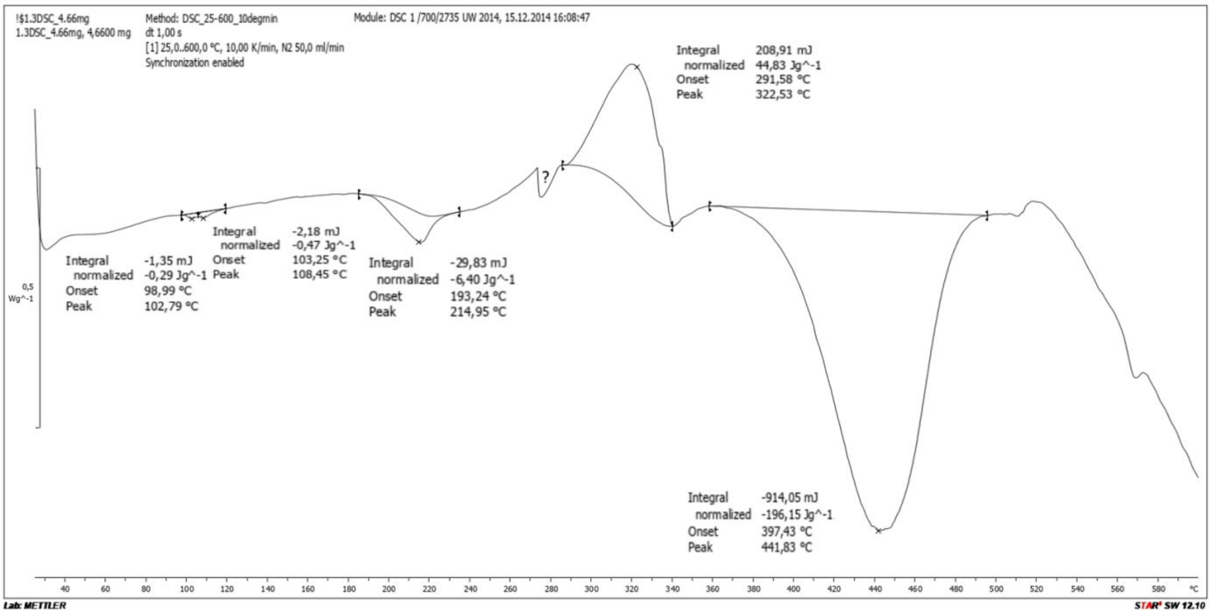

Figure S20 DSC curve of the cyclohexyl isocyanide the oligomerization product catalyzed by the $[Ni(TDA)(phen)H_2O]$

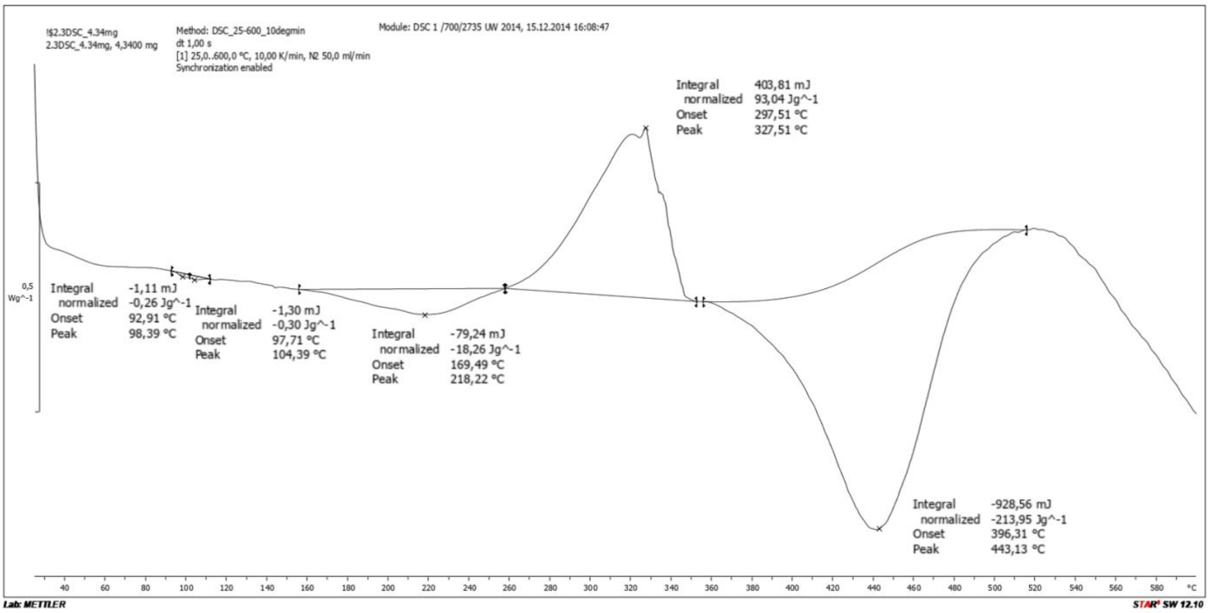

Figure S21 DSC curve of the cyclohexyl isocyanide the oligomerization product catalyzed by the $[Ni(TDA)(bipy)(H_2O)]{\cdot}4\ H_2O$

8. Simultaneous TGA/DSC thermal analysis

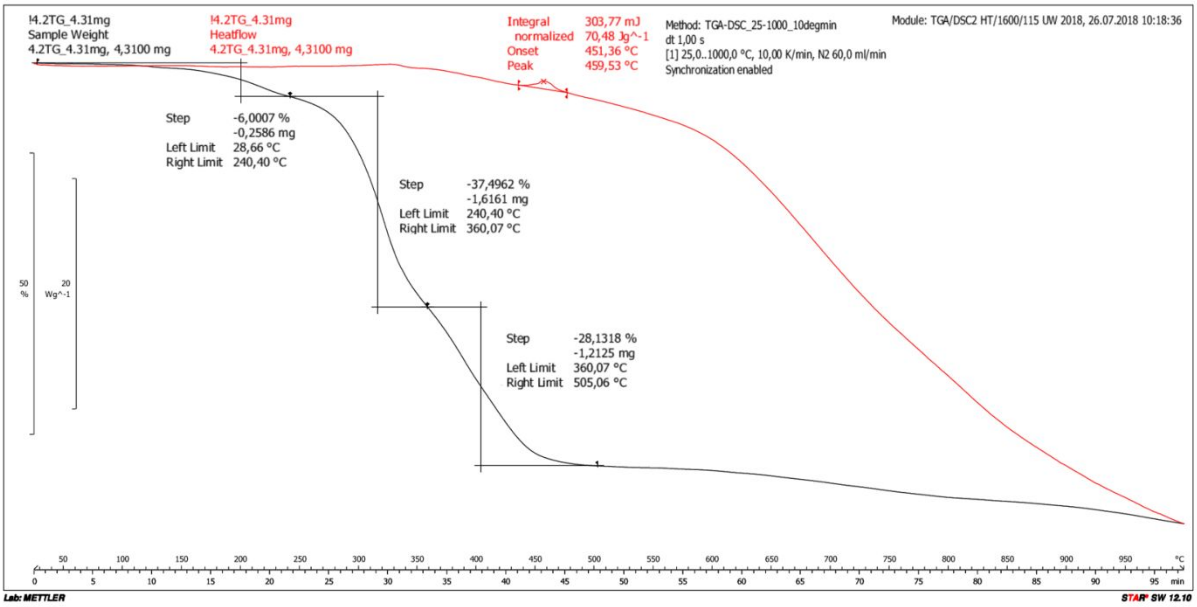

Figure S22 DSC/TGA curve of the cyclohexyl isocyanide the oligomerization product catalyzed by the [Ni(IDA)(H_2O)$_2$]

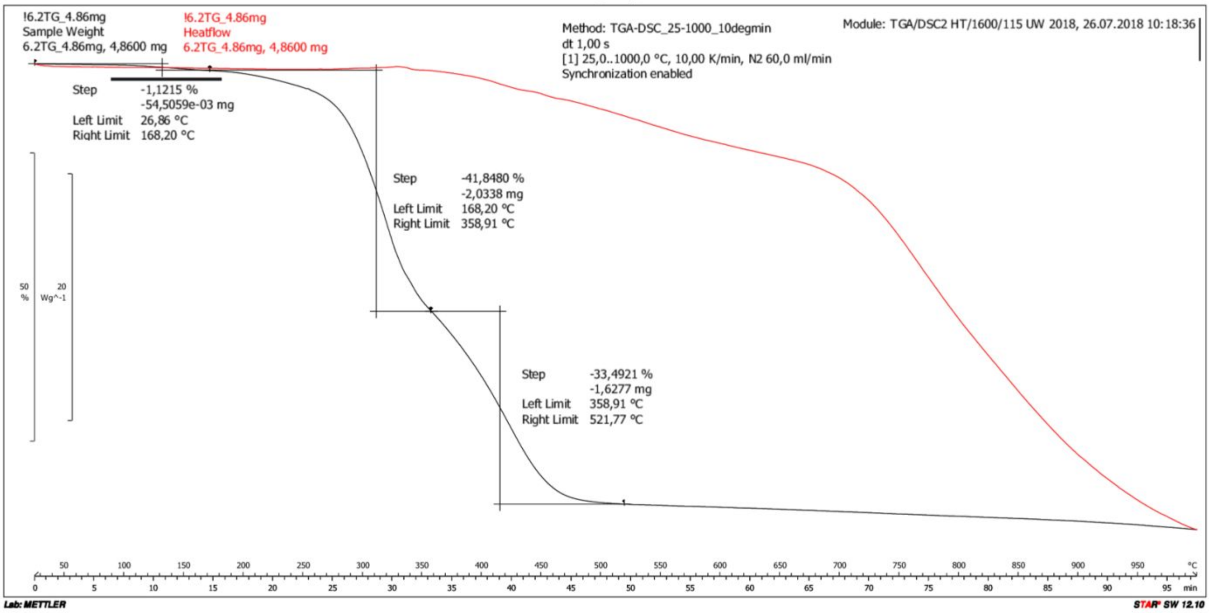

Figure S23 DSC/TGA curve of the cyclohexyl isocyanide the oligomerization product catalyzed by the [Ni(ODA)(H_2O)$_3$]·1.5 H_2O

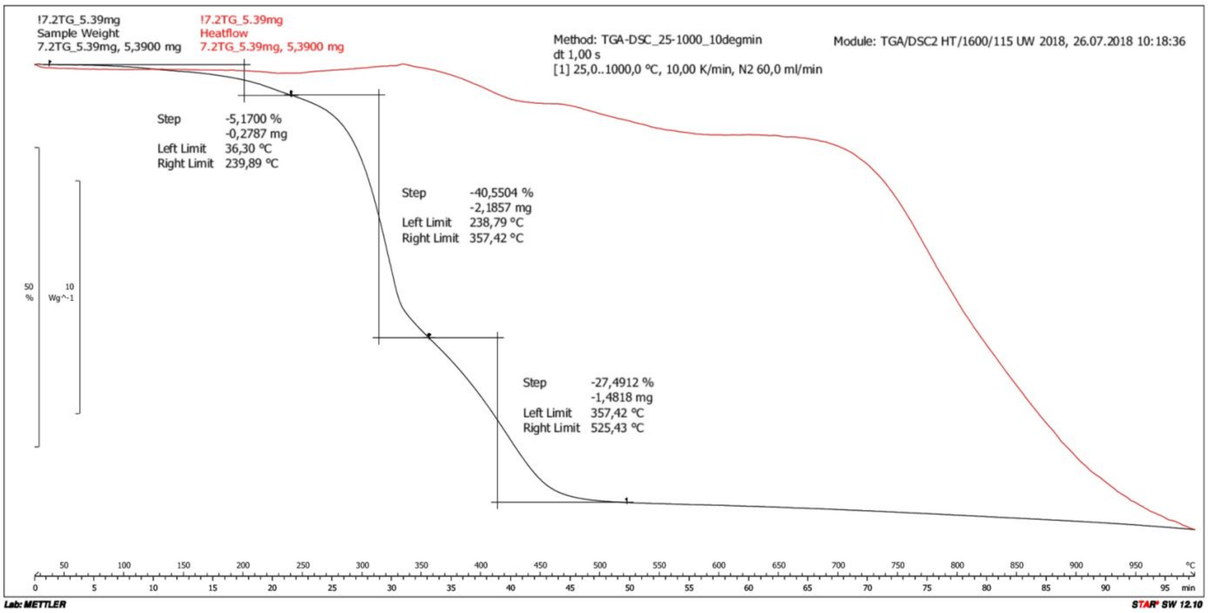

Figure S24 DSC/TGA curve of the cyclohexyl isocyanide the oligomerization product catalyzed by the [Ni(TDA)(H_2O)$_3$]

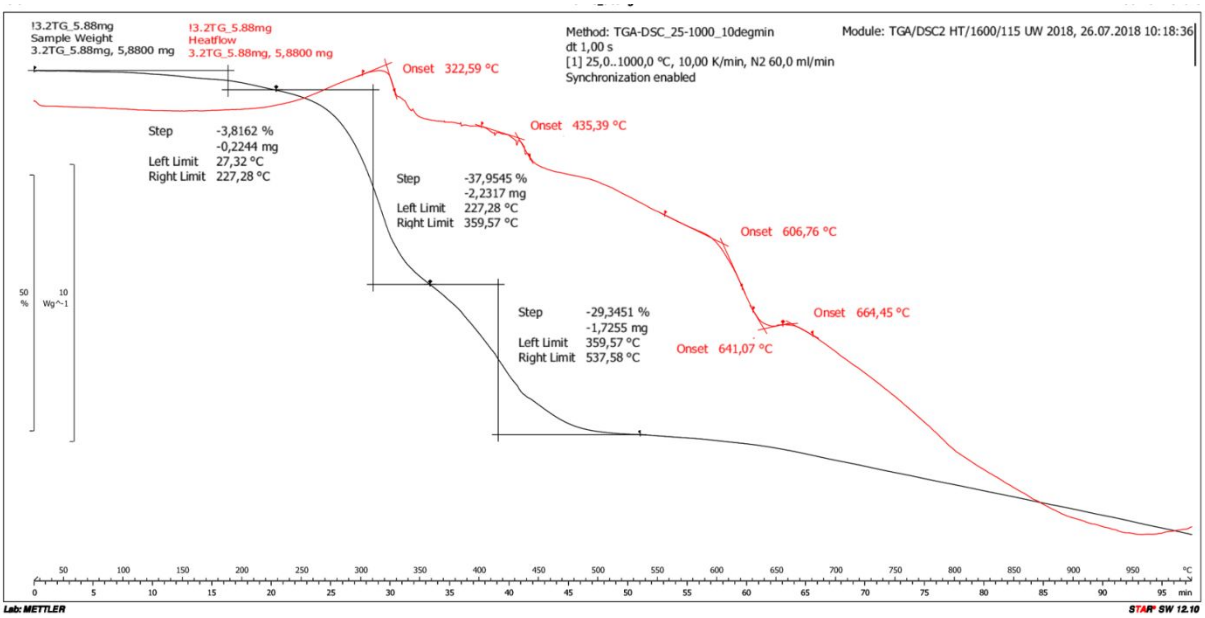

Figure S25 DSC/TGA curve of the cyclohexyl isocyanide the oligomerization product catalyzed by the [Ni(ODA)(phen)(H_2O)]·1.5 H_2O

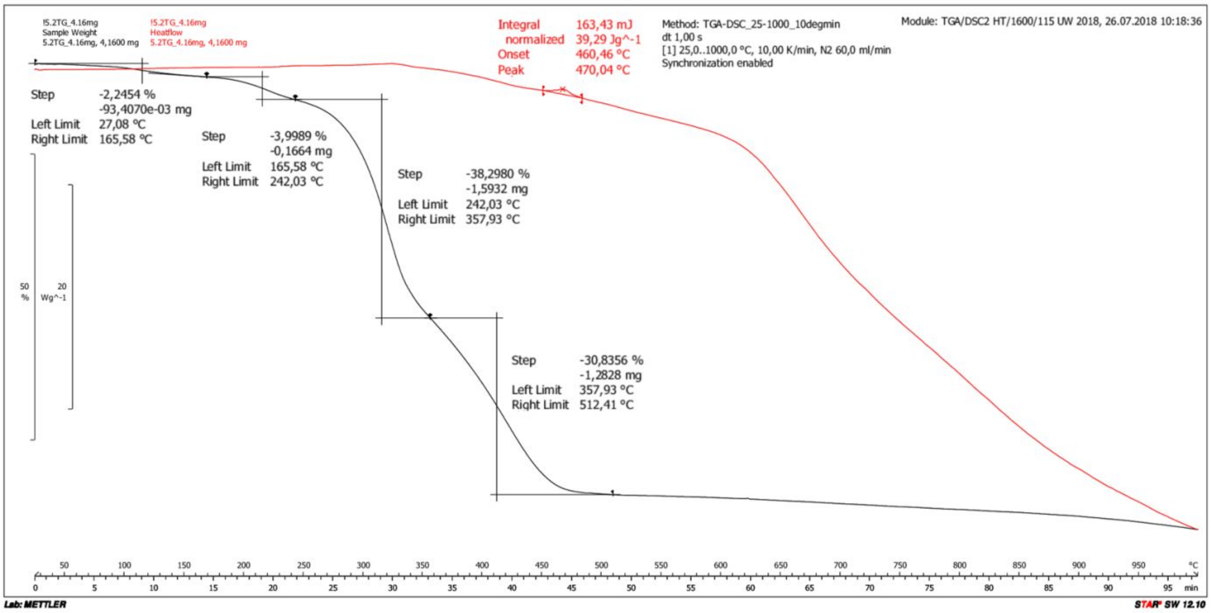

Figure S26 DSC/TGA curve of the cyclohexyl isocyanide the oligomerization product catalyzed by the [Ni(ODA)(bipy)(H_2O)]·2.5 H_2O

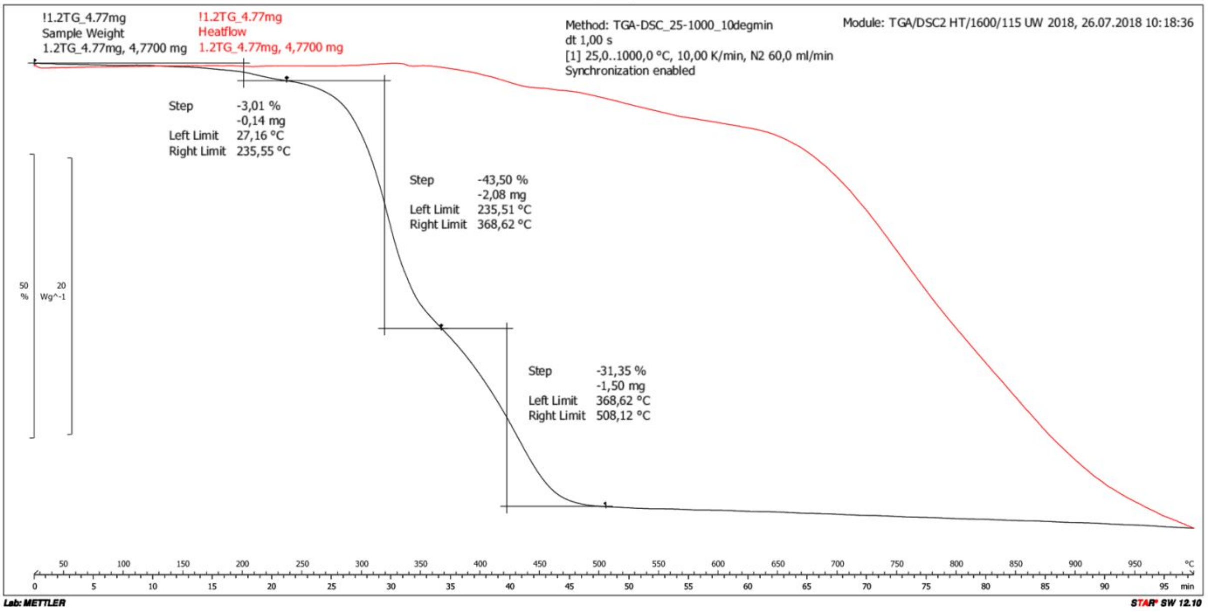

Figure S27 DSC/TGA curve of the cyclohexyl isocyanide the oligomerization product catalyzed by the [Ni(TDA)(phen)H_2O]

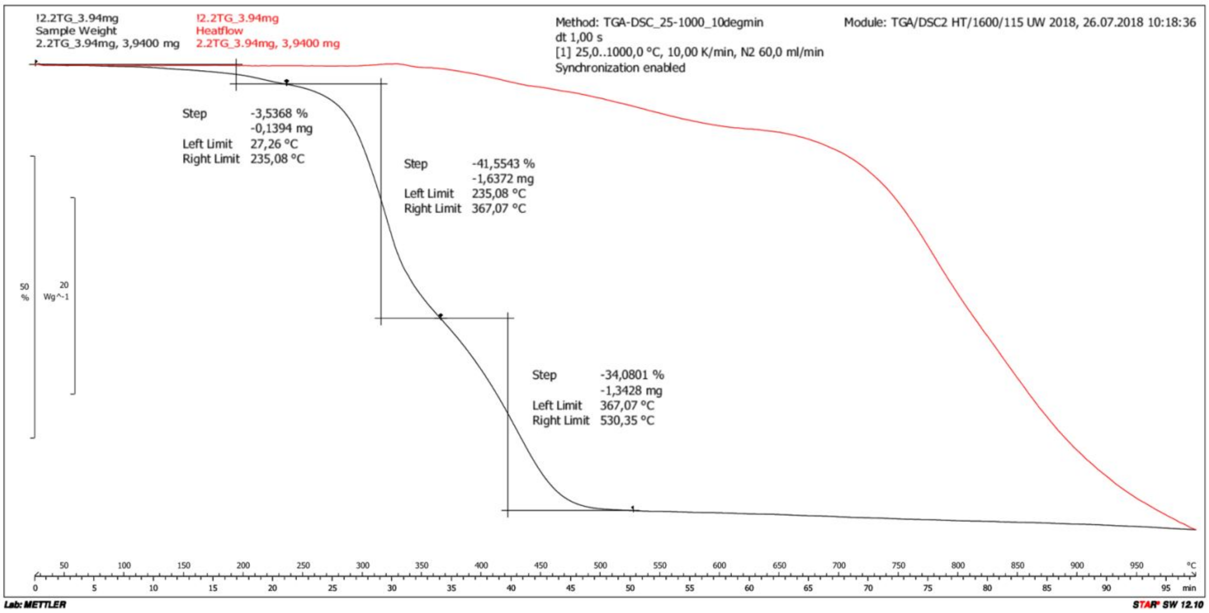

Figure S28 DSC/TGA curve of the cyclohexyl isocyanide the oligomerization product catalyzed by the [Ni(TDA)(bipy)(H_2O)]·4 H_2O
